# 2^e^ colloque Mayotte en Santé - Territoires défavorisés 18-20 septembre 2023 – Mayotte

**DOI:** 10.48327/mtsi.v4i2.2024.528

**Published:** 2024-06-24

**Authors:** Moncef MOUHOUDHOIRE, Karim ABDELMOUMEN, Éric PLEIGNET, Marie ÖNGÜN-ROMBALDI, Marine GAUBERT, Raïssa HOUMADI, Margot OBERLIS, Nicolas VIGNIER, Bernard CASTAN, Loïc EPELBOIN

**Affiliations:** 1 Nariké M’sada, Mamoudzou, 97600, Mayotte, France; 2 Unité des maladies infectieuses et tropicales, Centre hospitalier de Mayotte, Mamoudzou, 97600, Mayotte, France; 3 Association Oppelia - Addiction/Santé/Solidarité, POPAM (Plateforme de prévention, réduction des risques et de soin des addictions), Mayotte, France; 4 Fédération Addiction, Paris, France; 5 Institut toulousain des maladies infectieuses et inflammatoires - Infinity - Institut national de la santé et de la recherche médicale (Inserm), UMR 1291 - Centre national de la recherche scientifique (Cnrs), UMR 5051 - Université de Toulouse III, Toulouse, France; 6 Croix Rouge française, Cayenne, Guyane, France; 7 Hôpitaux universitaires Paris Seine-Saint-Denis, Hôpital Avicenne, Hôpital Jean Verdier, AP-HP, UFR SMBH, Université Sorbonne Paris Nord, IAME, Inserm UMR 1137, Bobigny, France; 8 Société de pathologie infectieuse de langue française, Paris, France; 9 Société française de lutte contre le sida, Nice, France; 10 Département de maladies infectieuses et tropicales, Centre hospitalier de Périgueux, Périgueux, France; 11 Unité des maladies infectieuses et tropicales, Centre hospitalier de Cayenne, Cayenne, France; 12 Centre d’investigation clinique Antilles Guyane, Inserm CIC 1424, Centre hospitalier de Cayenne, Cayenne, France

## Éditorial

Au cœur de la petite île de Mayotte, au Sud-Ouest de l’océan Indien, à environ 8 000 km de Paris, 11 000 km de Cayenne, 1 400 km de Saint-Denis de la Réunion et 700 km de Tananarive, dans les bâtiments récemment inaugurés du Pôle d’excellence rurale de Coconi, s’est déroulé du 18 au 20 septembre 2023 un colloque d’une ampleur et d’une qualité jusqu’alors inégalée. Ce colloque « Mayotte en Santé » a été organisé par l’association Nariké M’sada et la plateforme Oppelia de prévention et de soin des addictions à Mayotte (POPAM), grâce au financement de l’Agence régionale de santé (ARS) de Mayotte et du Centre hospitalier de Mayotte, et avec le soutien de la Société française de lutte contre le sida (SFLS), de l’Agence nationale de recherche sur le VIH/sida, les hépatites et les maladies infectieuses émergentes (ANRS/MIE), de la Fédération Addiction, de la Société de pathologie infectieuse de langue française (SPILF), et de la Société francophone de médecine tropicale et santé internationale (SFMTSI). Les échanges scientifiques se sont déroulés sur trois jours autour des thématiques suivantes : la santé sexuelle et reproductive (SSR), les maladies infectieuses émergentes (MIE) et les addictions. Le colloque a réuni 79 intervenants originaires de nombreux territoires : Mayotte, bien sûr, mais aussi La Réunion, Madagascar, la Guyane, la Martinique, la Guadeloupe et l’Hexagone, pour proposer plus de 50 interventions et tables rondes aux 723 participants en présentiel, et 534 participants en distanciel sur les trois jours. Soit un programme extrêmement riche.

Au cours de la session MIE, après une introduction du Pr Yazdan Yazdanpanah, directeur de l’ANRS/MIE et du Dr Bernard Castan, président de la SPILF, des thèmes passionnants se sont succédé, tels que : l’angiostrongylose nerveuse, la peste, la fièvre Q, la leptospirose, l’antibiorésistance, le paludisme, le tétanos, le béribéri, les risques d’émergence dans l’océan Indien. Ces questions ont été traitées sous plusieurs angles (celui du clinicien, du biologiste, de l’entomologiste et de l’épidémiologiste), et de façon large, puisqu’elles ont abordé la recherche d’agents pathogènes dans les eaux usées, mais aussi les conséquences de la pénurie d’eau, ou les campagnes de vaccination à Mayotte. Une session a été consacrée à l’échange d’expériences autour des équipes eau, hygiène et assainissement des Croix Rouge de Guyane et de Mayotte.

La session addictologie a été introduite par Marie Öngün-Rombaldi, déléguée générale de la Fédération Addiction et Naïra Meliava, directrice générale d’Oppelia. De nombreux thèmes ont été abordés, suscitant l’objet des débats passionnés : la justice résolutive de problèmes; l’éducation préventive (intervention précoce et conduites à risques); les troubles psychiatriques; la santé mentale; les conduites addictives; la présentation du dispositif SINTES (Système d’identification national des toxiques et des substances) de l’Observatoire français des drogues et des tendances addictives; le circuit d’analyse de produits et la veille sanitaire; le projet CheckNow (Évaluation du déploiement et de l’efficacité de l’analyse de drogues en France); la réalisation d’un diagnostic communautaire dans le cadre de l’implantation de la POPAM; la présentation des résultats préliminaires de « Chasse-marée - Étude de la chimique à Mayotte ».

Enfin, après une introduction de la session santé sexuelle par le Pr Françoise Barré Sinoussi, prix Nobel de physiologie ou médecine et présidente de Sidaction, et par le Pr Christine Katlama, professeure de maladies infectieuses à l’hôpital Pitié-Salpêtrière et présidente de l’AFRAVIH, de multiples sujets ont été abordés tels que les objectifs Onusida, les infections sexuellement transmissibles (IST) bactériennes, le VIH, les hépatites, les inégalités sociales de santé, la prophylaxie pré-exposition, le dépistage et les perspectives de la lutte contre le VIH/sida, l’épidémie de VIH dans les DROM/COM, le préservatif, les crises migratoires, les infections opportunistes, les échecs virologiques, les traitements et qualité de vie des personnes vivant avec le VIH (PVVIH), les actualités thérapeutiques, les réservoirs du VIH, la vie sexuelle des PVVIH, la prostitution, et l’IVG.

Le congrès a ainsi tenu ses promesses, mélangeant dans une approche multidisciplinaire (personnels associatifs, soignants, épidémiologistes, chercheurs), et par des interventions solennelles et des tables rondes, problématiques locales et globales, maladies tropicales négligées et agents pathogènes pandémiques, médecine, sciences humaines, psychologie, épidémiologie, addictologie, etc.

La qualité du fond comme de la forme fait espérer une nouvelle session en 2024, dans le 100^e^ département de France !

**Figure 1:**
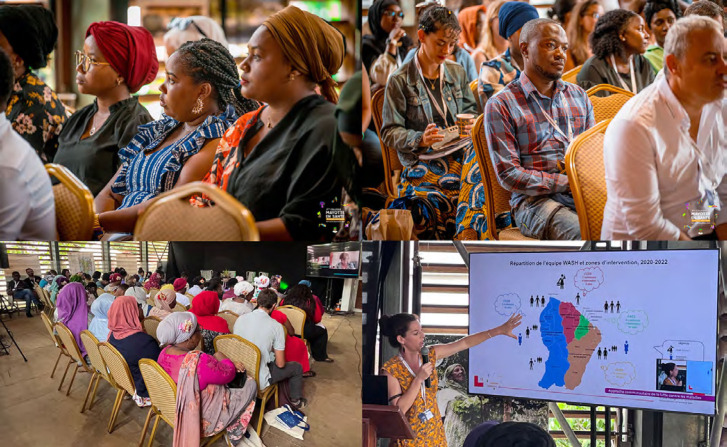
Photos du 2^e^ colloque Mayotte en Santé - Territoires défavorisés (crédit photo : haut droite et haut gauche : Instant Shoot©, bas droite et bas gauche : Loïc Epelboin) Photos of the 2nd Mayotte en Santé conference - Disadvantaged territories (photo credit: top right and top left: Instant Shoot©, bottom right and bottom left: Loïc Epelboin)

**Figure 1:**
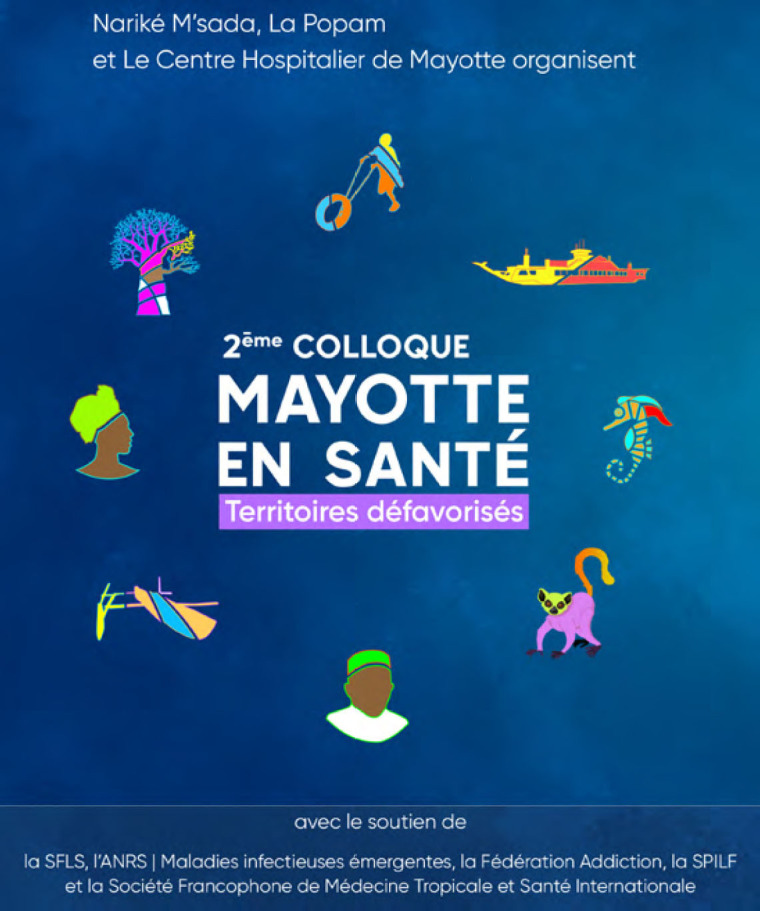
Affiche du 2^e^ colloque Mayotte en Santé - Territoires défavorisés (Réalisation : Association Nariké M'Sada) Poster of the 2nd Mayotte en Santé conference Disadvantaged territories ((Produced by: Association Nariké M'Sada

