# Supplementary material for: 2nd Mayotte en Santé conference - Disadvantaged territories - September 18-20, 2023 - Mayotte
Source: Med Trop Sante Int. 2024 Jun 24;4(2):mtsi.v4i2.2024.528. [Article in French] doi: 10.48327/mtsi.v4i2.2024.528 (PMC11292440; doi:10.48327/mtsi.v4i2.2024.528)
Supplement: Related texts

# Research article: Présentation du dispositif SINTES de l'Observatoire français des drogues et des tendances addictives (OFDT)

**Published:** 2024-06-24

**Authors:** Elodie Auzole

**Affiliations:** Dispositif TREND-SINTES, SAOME (Santé Addictions Outre-Mer), Saint-Denis de La Réunion

Le dispositif SINTES (Système d’identification national des toxiques et des substances) est porté par l’OFDT. Il existe depuis 1999 et compte 15 coordinations, dont deux en outre-mer : en Guyane et, depuis 2021, à La Réunion. SAOME (Santé Addictions OutreMer) a été désigné comme l’opérateur local du dispositif.

SINTES a trois missions : analyser et informer les usagers sur la composition des produits collectés, contribuer aux systèmes d’alerte sanitaire national (Signal drogues) et européen *(Early Warning System)* et documenter l’évolution des produits psychoactifs, leurs effets, leurs contextes d’usages ou leurs nouvelles compositions chimiques.

Il ne s’inscrit pas dans une démarche de réduction des risques immédiate puisqu’il est mobilisé pour analyser et collecter des substances le plus souvent après leur consommation par les usagers et ce, pour cinq motifs de collectes :

- s’il s’agit d’une substance non identifiée par le dispositif d’analyse de produits par chromatographie sur couche mince (CCM). À noter qu’à La Réunion, il n’existe pas encore ce type de dispositif d’analyse;
- si l’usager a subi des effets indésirables : réactions nocives après consommation d’un produit habituel, au contexte de consommation habituel;
- si l’usager a subi des effets inhabituels : par exemple, le produit a entraîné des sensations qui ne correspondent pas à celles couramment attendues avec celuici;
- si le produit a un caractère nouveau ou rare, comme des nouveaux produits de synthèse, un produit « classique », mais nouveau localement, de nouvelles présentations ou de nouvelles appellations, etc.; enfin, dans le cadre de la veille active de l’OFDT, si la coordination nationale ou régionale juge nécessaire le suivi d’un produit particulier. Le volet « Veille » est un système permanent d’information rapide qui s’inscrit dans une perspective de santé publique et cherche à détecter la présence de substances nouvelles ou inattendues dans les produits psychoactifs circulants sur le territoire.

Processus de collecte : l’usager apporte un produit à un collecteur formé par la coordination régionale, qui en évalue la pertinence. Le collecteur remplit un questionnaire avec l’usager et précise la situation avec lui puis envoie l’échantillon et le questionnaire au laboratoire. Un exemplaire du questionnaire est également envoyé à la coordination nationale qui interprètera les analyses que le laboratoire lui aura remis. L’ensemble est envoyé à la coordination régionale qui informera le collecteur. Ce dernier pourra alors contacter l’usager en l’informant de la composition du produit.

La documentation sur l’évolution des produits psychoactifs se traduit par une compilation et un croisement de différentes sources (les collectes SINTES, les données TREND (Tendances récentes et nouvelles drogues), les laboratoires partenaires, les réseaux des Centres d’évaluation et d’information sur la pharmacodépendance-addictovigilance (CEIP-A) et les données policières). Ce travail donne lieu à la publication du Point SINTES de l’OFDT qui recense toutes les nouvelles évolutions de l’année.

# Research article: Chasse-Marée : étude de la « chimique » à Mayotte

**Published:** 2024-06-24

**Authors:** Damien A. DEVAULT, Victoire COTTEREAU, Jean-Michel GAULIER, Amélie Daveluy, Alexandre PEYRÉ, Camille RICHEVAL, Alexandr GISH, Éric PLEIGNET

**Affiliations:** Centre universitaire de formation et de recherche de Mayotte, Mayotte, France Laboratoire Espace-Dev, France; Laboratoire du Centre hospitalier régional universitaire de Lille, France; Centre d’évaluation et d’information sur les pharmacodépendances - addictovigilance (CEIP-A), Service de pharmacologie médicale, Université de Bordeaux.; Association Oppelia – addiction / santé / solidarité, POPAM (Prévention et de soin des addictions), Mayotte, France

Depuis le début des années 2010, les cannabinoïdes de synthèse (CS) sont consommés à Mayotte, sous la formulation de « chimique » : une cigarette dont le végétal (tabac, pour l’essentiel) a été plongé dans un mélange de solvants et de substances psychoactives. Au problème sanitaire identifié de 2012 à 2020 (altération de la conscience, voire overdoses en pleine rue), a succédé une invisibilisation de cette consommation dans les sources sanitaires et judiciaires. Le programme de recherche CHASSE-MARÉE a voulu faire le point.

Il s’est articulé autour de trois tâches : (1) identifier les molécules composant les cigarettes (substrat végétal, produits de coupe, CS) et leur métabolisation; (2) définir le profil des consommateurs et leurs habitudes de consommation; (3) vérifier s’il est possible d’appliquer l’approche dite de « l’épidémiologie des eaux usées » pour les CS, nonobstant leur nombre et leur diversité chimique.

En pratique, huit campagnes mensuelles de collecte furent organisées, chacune durant une semaine, sans limite maximale du nombre d’échantillons et de questionnaires auprès des consommateurs.

Dans le cadre du programme, des consommateurs (dont certains sont des « cuisiniers », c’est-à-dire ceux qui font la préparation avant consommation) ont fourni des échantillons de poudre. La moitié des échantillons ne comportait qu’un seul principe actif. Les résultats laissent à penser qu’au moins une partie des « cuisiniers » ne procède qu’à l’imbibition finale d’une poudre fournie par des grossistes.

Quatre produits de coupe ont été identifiés : si ces résultats laissent entrevoir plusieurs hypothèses, ils n’ont pas confirmé la réputation de la « chimique » d’être composée de produits ménagers nocifs.

L’analyse révèle une très nette volatilité du marché : d’un mois à l’autre, la composition différait fortement. Parmi les 11 molécules identifiées dans notre étude, 6 le furent pour la première fois en France ou même en Europe. La métabolisation de la plupart des molécules n’est pas connue. Les plus observées furent MDMB-4en-PINACA et MDMB INACA alors que l’analyse au cours du présent programme de « chimique » saisie dans le domaine carcéral en 2018 souligne la présence de 4F-ABINACA et 4F-MDMB-BICA. Les premières analyses statistiques réalisées à partir de 121 questionnaires établissent un profil moyen du consommateur. Il s’agit d’un homme dans la trentaine, de nationalité française ou comorienne et au niveau de vie précaire (chômeur très peu qualifié, sous le seuil de pauvreté) donc similaire à l’ensemble de la population résidente de Mayotte. Il vit en famille en logement précaire (tôle) avec eau et électricité, consomme au minimum 10 fois dans le mois un produit appelé « chimique » dont il ne sait pas dire la composition ni l’origine, mais qui lui permet d’oublier ses problèmes. Les effets indésirables ne poussent qu’un tiers des 121 consommateurs interrogés à souhaiter arrêter.

La métabolomique des molécules n’est élucidée que pour deux d’entre elles à cette heure. Néanmoins, la « chimique » devient de plus en plus cachée à mesure que l’ostracisme contre les consommateurs croît et de leur avancée en âge. Enfin, toute une population consommatrice nous échappe peut-être, mais serait perçue par le suivi des eaux usées. Par son ampleur, sa fréquence, sa pluridisciplinarité, le programme CHASSE-MARÉE, financé par l’Agence régionale de santé de Mayotte et appuyée par la MILDECA et l’ODFT, renouvelle l’éclairage sur la composition de la « chimique » et sur ses consommateurs, et contribue à une meilleure connaissance des CS.

# Research article: Le circuit d'analyse de produits et la veille sanitaire – Perspectives du colloque Mayotte en Santé

**Published:** 2024-06-24

**Authors:** Nicolas GERSTEL, Elodie AUZOLE, Perrine ROUX, Alexandre PEYRE, Quentin GORRIAS, Marie ÖN-GÜN-ROMBALDI

**Affiliations:** Association Oppelia – addiction / santé / solidarité, POPAM (Plateforme Oppelia de prévention et de soin des addictions), Mayotte, France; Dispositif TReND-SINTES, SAOME - santé addictions Outre-Mer, Saint Denis de La Réunion; Aix Marseille Université, Inserm, IRD (Institut de recherche pour le développement), SESSTIM (Sciences économiques & sociales de la santé & traitement de l’information médicale), ISSPAM (Institut des sciences de la santé publique), Faculté de médecine, 27 Bd Jean Moulin, 13385 Marseille Cedex 5, France; Centre d’évaluation et d’information sur les pharmacodépendances - addictovigilance (CEIP-A), Service de pharmacologie médicale, Université de Bordeaux.; Association Réseau Oté, Saint Paul, La Réunion. Fédération Addiction, Paris, France

La table ronde « Le circuit d’analyse de produits et la veille sanitaire – Perspectives » faisait suite à la présentation de l’étude « Chasse-Marée » réalisée sur le territoire de Mayotte. Elle avait comme objectif de présenter les dispositifs d’analyse de produits existants, aussi bien du point de vue de la veille sanitaire que de la réduction des risques et des dommages (RdRD) directement auprès des consommateurs.

Ainsi, une présentation de l’étude « CheckNow », en cours de réalisation en métropole a été faite. Il s’agit d’un projet de recherche communautaire visant à démontrer l’efficacité de l’analyse de produits à visée de RdRD auprès des usagers de drogues. La présentation a mis en exergue les premiers résultats d’un questionnaire explorant les attentes et les perceptions des intervenants proposant l’analyse de drogues comme outil de RdRD. Ce projet montre aussi la pluralité des dispositifs existants en termes d’analyse de produits en direct -chromatographie sur couche mince (CCM), spectrométrie, chromatographie en phase liquide à haute performance (HPLC)-, permettant de se servir de cette analyse comme support à la réduction des risques avec les usagers au travers de résultats qualitatifs et/ou quantitatifs des produits allant être consommés.

La présentation du dispositif SINTES porté par l’Observatoire français des drogues et des tendances addictives (OFDT) a mis en lumière la complémentarité de ce dispositif, l’analyse de drogues ayant un objectif de RdRD proposé par des structures de réduction des risques.

Le département de Mayotte ne bénéficiant pas encore de ces dispositifs a profité, au travers de l’étude « Chasse-marée », des prémices de ce qui pourrait être développé sur le territoire. Le travail de terrain réalisé par la POPAM a aussi montré des besoins.

Les consommateurs rencontrés tout au long de l’étude semblent montrer une connaissance relative des produits qu’ils consomment et des risques associés. L’analyse de ces produits montre une grande « volatilité » des molécules et donc un besoin de suivre au plus près l’évolution de la composition des préparations « locales ». Ce besoin relève aussi de demandes émises par les consommateurs lors du travail de rue effectué par les professionnels de la POPAM depuis deux ans. Ces demandes portent sur une meilleure compréhension et/ou connaissance des produits consommés au-delà de la « chimique ». Un autre besoin a été repéré lors des interventions en milieu festif, auprès d’un public *a priori* « plus inséré » dont les conduites à risque (primo-consommations de nouveaux produits de synthèse (NPS), conduites sexuelles à risques, surdosage, usage détourné de médicaments…) semblent exacerbées sur l’île par rapport à des contextes équivalents sur d’autres territoires. Ce public souhaite pouvoir bénéficier d’un résultat et d’informations adaptées immédiates concernant les produits qu’ils vont consommer dans la soirée.

Ces observations montrent le besoin de développer le dispositif SINTES sur le territoire mais également de bénéficier d’un dispositif d’analyse mobile pour enrichir le travail de RdRD réalisé par la POPAM. Des échanges entre le public et les participants à cette table ronde ont ensuite exploré les différentes manières d’y parvenir et de préciser les formes que cela pourrait prendre.

# Research article: Programmes probants et compétences psychosociales (CPS) en milieu scolaire

**Published:** 2024-06-24

**Authors:** Quentin Gorrias

**Affiliations:** Association Réseau Oté, Saint Paul, La Réunion.

Le programme PRIMAVERA est un programme de prévention des conduites à risques et addictives développé par l’association OPPELIA.

L’association Réseau Oté ! est porteuse de ce programme depuis trois ans. L’association met en œuvre ce programme sur deux communes de l’île de la Réunion, ce qui représente 125 classes.

Il s’agit d’un programme reposant sur le développement des compétences psychosociales des élèves scolarisés entre le CM1 et la 5^e^, c’est-à-dire leur capacité à maintenir un état de bien être subjectif qui leur permet d’adopter un comportement approprié et positif à l’occasion d’interactions avec les autres, leur culture et leur environnement. La compétence psychosociale joue un rôle important dans la promotion de la santé dans son acception large renvoyant au bien-être physique, psychique et social.

Ce programme s’appuie également sur le vécu subjectif ou observé des jeunes afin de se rapprocher de la réalité des expériences que les élèves peuvent traverser.

Les critères d’évaluation d’un programme probant sont les suivants :

- le programme doit se composer de plusieurs séances dans l’année;
- le programme doit participer à la remobilisation des CPS;
- les professionnels de la prévention doivent collaborer avec l’ensemble des adultes environnant les élèves;
- le programme doit être évalué scientifiquement;
- il doit inclure une approche expérientielle.

Le programme PRIMAVERA est un programme « probant ». Cela signifie que le contenu de ce programme a bénéficié d’une évaluation scientifique par l’INSERM qui a validé l’ensemble de ses composantes. L’une des composantes essentielles, condition *sine qua non* de sa mise en œuvre, est le fait d’impliquer en tant que co-animateur ou co-animatrice et ce de manière systématique, un professionnel du champ de l’Éducation nationale dans les séances de prévention, sous forme d’ateliers auprès des élèves. Cela participe également au caractère « transférable » du programme. En effet, l’une des finalités de ce programme est de rendre autonome les établissements scolaires dans sa mise en œuvre. Cela revient à dire que les équipes de chargés de prévention pourront à terme se retirer pour que les enseignants puissent mener seuls les ateliers face aux élèves. Il vise la prévention des conduites addictives par le développement des compétences psychosociales des élèves dans une approche qui se veut participative et ludique. C’est pourquoi, il convient que les futurs co-animateurs et co-animatrices soient formés en amont des ateliers de prévention auprès des élèves.

L’expérience nous a montré qu’il est primordial que l’ensemble des parties prenantes soit impliqué en amont de la mise en œuvre.

Ainsi ARS, communes, rectorat, département et association porteuse doivent tisser un lien partenarial fort sur l’ensemble des composantes du programme afin de faciliter sa mise en œuvre. Ce programme représente un réel changement de paradigme pour les acteurs de l’Éducation nationale en les impliquant sur la question de la formation, de l’animation d’atelier, de la coordination territoriale. De ce fait, il est fondamental que chacun des acteurs mesure son degré d’implication.

Afin de s’assurer d’un impact global, le programme PRIMAVERA a pour but d’impliquer les élèves aux côtés des enseignants et des parents. À cette fin, des outils de liaison sont utilisés, tels que des livrets primaires, collèges et co-animateurs. Cela participe à faire perdurer l’action au-delà de la présence des chargés de prévention lors des séances de prévention.

# Research article: Réalisation d'un diagnostic communautaire dans le cadre de l'implantation de la POPAM (plateforme Oppelia de prévention et soin des addictions à Mayotte)

**Published:** 2024-06-24

**Authors:** Teresa Silva, Thibault RIBEAUCOURT, Anne LAURENT

**Affiliations:** Association Oppelia – addiction / santé / solidarité, POPAM (Prévention et de soin des addictions), Mayotte, France; Association Pas de Côté en santé, Alès, France

La plateforme Oppelia de prévention et de soin des addictions à Mayotte (POPAM), a été ouverte au public en janvier 2022 après une période de préfiguration de plusieurs mois (juillet à décembre 2021). Dans le même temps, un diagnostic communautaire a été initié, dont les objectifs étaient de mieux connaître le territoire d’implantation de la structure et d’être connu de celui-ci, d’appréhender les modes de consommations des usagers et d’analyser les représentations concernant les addictions des personnes vivant sur le territoire. L’ensemble de l’équipe a été impliqué dans l’élaboration et la réalisation de ce travail. Celui-ci s’est déroulé en plusieurs étapes : le choix du territoire du diagnostic, des informations pertinentes, des méthodes de recueil, puis l’élaboration des outils et le recueil d’informations. Le diagnostic s’est déroulé à Tsingoni, lieu d’implantation de la POPAM. Ce choix est lié aux caractéristiques de Tsingoni proches des caractéristiques sociodémographiques globales de Mayotte. Le diagnostic était aussi une opportunité de se faire connaître des habitants. Quatre méthodes de recueil ont été déployées : un diagnostic en marchant (cette méthode consiste en une déambulation dans l’espace public et un recueil d’informations à l’aide d’une grille d’observation) visant à repérer et décrire les lieux de consommation, des entretiens individuels et des entretiens collectifs auprès d’usagers pour appréhender les motifs de consommation, les usages, les produits, le lien avec la religion et enfin, un « *World Brachetti »* (adaptation de la méthode du *World café* au contexte mahorais, c’est-à-dire dans le cadre d’un repas communautaire) avec des acteurs du territoire pour analyser les représentations des addictions et les besoins en lien avec les missions de la POPAM.

Le diagnostic en marchant s’est déroulé dans plusieurs communes : Tsingoni, Combani, Mirereni et Mroalé. Au total, 19 lieux de consommation ont été repérés et décrits. Les entretiens et groupes ont mobilisé 60 personnes, usagers ou non. Les résultats produits ont permis de décrire les motivations à consommer, le fonctionnement des groupes d’usagers et les règles de solidarité et de contribution qui les régissent. L’invisibilité des femmes dans ces espaces et plus généralement sur les questions de consommation a été appréhendée. Le rapport à la religion et à la culture a été abordé avec l’identification de « tiraillements » entre les croyances, et les règles liées à la religion musulmane dans un contexte culturel qui inscrit cependant la consommation de produits dans les rites et pratiques. Les représentations des consommateurs pour la population rencontrée désignent essentiellement les jeunes comme étant concernés. Pourtant, la file active de la POPAM est âgée en moyenne de 38 ans. Ce décalage peut être l’expression d’une mise à distance de la consommation chez les adultes, mais aussi l’expression d’une invisibilité des consommateurs plus âgés dans l’espace public ou d’un moindre recours des plus jeunes aux structures d’aide.

Ce travail a tout autant permis le recueil d’informations utiles pour réajuster le projet de la POPAM que l’expérience, par l’équipe d’un projet collectif. Le diagnostic communautaire est une stratégie pertinente pour aller à la rencontre des acteurs d’un territoire et amorcer une démarche plus large de réflexion et d’élaboration de solutions concertées, que ce soit en matière de messages de prévention, ou de mise à disposition de matériel de réduction des risques. L’implantation sur l’île de deux nouveaux sites de la POPAM devrait permettre d’approfondir encore le diagnostic issu d’une démarche communautaire en santé, et d’ajuster, lorsque cela est possible, des réponses aux attentes et besoins d’une population, dans un contexte de bouleversements démographique, social, économique et culturel.

# Research article: Troubles psychiatriques et conduites addictives : parcours de soin, de vie et rétablissement

**Published:** 2024-06-24

**Authors:** Naïra MELIAVA

**Affiliations:** Association Oppelia – addiction / santé / solidarité, Paris, France

L’enjeu pour les cliniciens est trop souvent limité par l’approche causale, dont le modèle sous-jacent reste la pharmaco-psychose :

- des troubles psychiatriques induisant une addiction, soit par automédication du trouble psychiatrique sous-jacent, soit par des comportements à risque, désinhibition, impulsivité, abus de substance cités dans le tableau clinique;
- des troubles addictifs induisant ou prolongeant des symptômes/troubles psychiatriques (cannabis et psychose, alcool et dépression).

Sans appréhender cette étroite liaison entre addictologie et psychiatrie, nous faisons émerger des patients « ni-ni », qui ne sont pris en charge ni par les structures de soins psychiatriques, ni par les structures addictologiques, ne correspondant jamais aux cadres prévus.

L’approche clinique des situations de double diagnostic nécessite une mise en suspens du jugement causal du praticien sur les troubles pour la mise en place de soins intégrés. L’étude ISADORA, une recherche prospective financée par la Commission européenne, sur le parcours des patients présentant une pathologie psychiatrique grave et une dépendance à un produit psychoactif a été réalisée entre 2002 et 2005 sur 7 sites européens auprès de 350 personnes avec une comorbidité psychiatrique associée à des conduites addictives. Cette étude a démontré que le système de soins n’arrive pas à s’adapter à une personne présentant une pathologie duelle. Il est essentiel pour cela de s’interroger sur le projet de vie de la personne pour ensuite y inscrire les soins nécessaires. Proposer l’ancrage de l’accompagnement dans des réponses concrètes en termes d’hébergement, de traitement, d’aides matérielles et financières, permet de soulager la détresse sociale, de ramener à la réalité des histoires de vie qui s’en marginalisent, de restaurer l’inscription symbolique de la personne dans le tissu social. En cela, la transdisciplinarité a des effets sur le psychisme et peut prévenir l’aggravation de troubles ou le basculement dans la pathologie. Les équipes sont ainsi des « tuteurs de résilience », en mesure de porter un diagnostic de mal-être psychique et social; ils ont un savoir et des modes d’intervention complémentaire à la dimension purement médicale. Les centres de soins d’accompagnement et de prévention en addictologie (CSAPA) et les centres d’accueil et d’accompagnement à la réduction des risques pour usagers de drogues (CAARUD) peuvent répondre de façon adaptée aux besoins psychiques de certains publics. Par exemple, des dispositifs d’accueil à bas seuil pour des publics précarisés qui s’inscrivent dans l’évitement d’une relation d’aide. Ils peuvent favoriser par l’accueil inconditionnel qu’ils proposent, un environnement favorable, un apaisement des tensions, une expression des consommations et une réassurance de la personne et, *in fine,* permettre de créer des liens avec la psychiatrie et d’articuler les parcours.

# Research article: Mettre la prévention des conduites à risques sur deux pieds : éducation préventive et intervention précoce

**Published:** 2024-06-24

**Authors:** Naïra MELIAVA

**Affiliations:** Association Oppelia – addiction / santé / solidarité, Paris, France

Il est impératif de sortir des impasses de la prévention conventionnelle telle que celleci a été développée durant de nombreuses années. Le champ des addictions est pétri de représentations contradictoires, confuses, de messages très généraux ou peu adaptés qui ne favorisent pas des actions cohérentes.

En effet, sous le prisme de l’addiction en tant que maladie ou fléau, les modalités d’intervention se sont résumées à la prévention par les dangers ou celle de l’interdit. Toutes deux ont démontré leur inefficience par la négation de la part de satisfaction et d’adaptation des conduites addictives et des capacités d’autocontrôle du public ciblé.

La prévention intervient à contretemps (quand le problème ne se pose pas) et à contre-courant (alors que l’usager en tire des bénéfices). Si soigner est contrôler les souffrances provoquées, prévenir devrait s’intéresser surtout aux satisfactions procurées (plaisir, soulagement, socialisation) et viser à mieux maîtriser les risques.

Trois impératifs se dégagent en faveur du changement de paradigme :

- élaborer de nouvelles représentations partagées, un langage commun et une démarche qui tienne compte de l’expérience de chacun. Co-construire un savoir qui intègre les savoirs expérientiels et les données scientifiques;
- construire un projet éducatif qui permette de le transmettre, comme, par exemple, apprendre le bon usage des objets et des techniques (entre autres, les produits psycho-actifs ou les réseaux sociaux);
- faire émerger de nouvelles pratiques.

Pour le mettre en œuvre, nous disposons des moyens suivants :

- apprendre à se servir des ressources individuelles et collectives pour des choix éclairés;
- ne pas diffuser uniquement de l’information, mais proposer un dispositif relationnel et d’implication. Toutes les méthodes ne se valent pas pour animer des séances-actions collectives;
- les approches de promotion de la santé constituent le socle des programmes probants, basés sur les compétences psycho-sociales et doivent être dans une démarche évaluative de leurs impacts;
- les approches de réduction des risques sont totalement intégrées aux interventions;
- les approches associent les familles et les acteurs du territoire afin de favoriser la promotion de la santé, la réduction des risques et valoriser l’expérience.

Prévenir ne se limite pas à diffuser des messages, c’est aussi intervenir avant la demande d’aide. Développer l’intervention précoce est une condition d’efficacité de la prévention. L’intervention précoce est une stratégie d’action entre la prévention et l’accès aux soins, dont l’objectif est de raccourcir autant que possible le délai entre l’apparition des premiers signes des troubles et la mise en œuvre de traitements ou d’accompagnements adaptés. Elle est une démarche communautaire en santé. Elle se décompose en quatre phases :

- phase 1. Promouvoir et renforcer un environnement favorable : aller au plus près des personnes en situation de risque, dans leur contexte;
- phase 2. Le repérage : aider à réaliser le repérage de ces personnes et les aider à gérer leur vulnérabilité;
- phase 3. L’évaluation : permettre de mieux appréhender des difficultés identifiées pour envisager de mieux les orienter vers les dispositifs adaptés;
- phase 4. L’accompagnement : offrir dans le délai le plus court, des services qui prennent en compte les compétences fragilisées.

Le rôle du politique est déterminant pour entériner ces changements d’approche et reconnaître réellement la prévention à sa juste valeur. Celui des acteurs l’est tout autant pour ouvrir des voies nouvelles et montrer leur efficience.

# Research article: Santé mentale et addictions : pourquoi et comment dépasser le modèle biomédical ?

**Published:** 2024-06-24

**Authors:** Alain MOREL

**Affiliations:** Psychiatre addictologue, laboratoire de recherche Oppelia – addiction / santé / solidarité, Paris, France

*« Si l’on y prend garde, ¡’Afrique risque de ne plus se voir que par les yeux des autres et de jeter sur elle-même un regard pétrifiant »» (Aimé Césaire, discours de Dakar, 1966).*

La consommation de substances psychoactives non prescrites (stupéfiants, alcool, médicaments, tabac) par des personnes ayant des troubles psychiques est un phénomène croissant, à Mayotte comme en Métropole. Il pose des difficultés conceptuelles et pratiques aux équipes de soin. Il interroge les représentations de la maladie mentale et celles de l’addiction, et révèle l’inadaptation du modèle biomédical qui le réduit à une « comorbidité » de deux « maladies du cerveau » affectant des individus.

L’approche globale, intégrant les dimensions socio-culturelle, psychologique et biologique, apporte un regard différent où la maladie mentale est une souffrance et la consommation de substances psychotropes marchandisées une tentative de gestion de cette souffrance.

Avec leur diversité d’intensité et d’accessibilité, les drogues s’inscrivent dans toutes les activités humaines. Elles peuvent servir à se divertir, se soulager, se socialiser, et, transitoirement, à s’apaiser, s’adapter et s’émanciper. Mais leur usage expose aussi à des risques : toxicité, perte de contrôle, dépendance, dommages médicaux et sociaux… Elles deviennent alors des cofacteurs de détérioration de la santé globale parmi d’autres déterminants : le stress, la précarité, les psycho-traumatismes, le déclassement, l’isolement, la violence, la stigmatisation, l’ennui, la dépression, l’hyperactivité, le développement du marché des drogues.

Ainsi, la vulnérabilité croissante à la souffrance psychique et aux addictions trouve sa source dans ce qui détériore nos liens sociaux. C’est donc là que se trouvent aussi les réponses : la réduction des inégalités, l’inclusion sociale, l’éducation et la promotion de la santé, la coopération, etc.

Le renforcement du pouvoir d’agir de la personne (pour son autonomie) et la qualité des relations sociales (pour se relier et coopérer) sont les clés du rétablissement des personnes et des transformations sociales nécessaires dans leur environnement.

Toutes les modalités d’intervention ne se valent pas pour poursuivre ces objectifs. Selon nous, deux réunissent les valeurs qui favorisent des pratiques coopératives et inclusives : la réduction des risques et la démarche communautaire. La démarche communautaire en santé entreprise par la POPAM à Tsingoni montre tout le potentiel de ce type de mobilisation pour la collectivité.

Plus que tout autre, le contexte mahorais nous invite à la créativité. Il est spécifique tant dans ses dimensions communautaires et culturelles, qu’au regard des tensions sociales, des consommations de substances psychoactives qui s’y développent et de la précarité des populations et des services de santé. Inventer des réponses adaptées commence par des alliances : entre les équipes psychiatriques et d’addictologie, mais aussi avec les usagers et les personnes vivant sur l’île. Il s’agit de reconnaître et d’impliquer toutes les parties de la communauté sociale, de donner la parole, partager et associer tous les savoirs dans un langage commun.

Bien évidemment, une telle dynamique repose sur des expérimentations concrètes, des « petites » réalisations, de la convivialité et de la confiance. et sur l’appui des autorités de santé.

# Research article: Justice résolutive de problèmes (JRP) et addictions : orientations et préconisations

**Published:** 2024-06-24

**Authors:** Eric PLEIGNET, Marine GAUBERT

**Affiliations:** Association Oppelia – Addiction / Santé / Solidarité, POPAM (Prévention et de soin des addictions), Mayotte, France; Marine Gaubert, Fédération Addiction, Paris.

« Née aux États-Unis, la première expérimentation de la JRP trouve son origine dans un quartier de la ville de Miami extrêmement dégradé par une épidémie de crack en 1989. Les magistrats organisèrent des réunions avec les citoyens locaux et les institutions pour aboutir ensemble à un modèle de justice pragmatique visant à cibler, puis traiter, l’ensemble des problèmes causant et causés par l’épidémie de crack » 11Les conditions scientifiques de l’efficacité des JRP pour la prise en charge des délinquants souffrants de toxicomanie. Enjeux et réponses. Sous la direction de Nathalie Roret. 2021 [accédé 26/03/2024].https://www.dro-gues.gouv.fr/le-rapport-de-synthese-sur-les-conditions-scientifiques-de-lefficacite-des-juridictions-resolutives.

Depuis quelques années, les domaines de la santé et de la justice expérimentent la manière dont une décision de justice peut engager des personnes multirécidivistes dans un parcours thérapeutique. Des éléments de solution ont ainsi pu être tirés d’expériences menées dans plusieurs départements. À partir de l’expertise de professionnels et de structures en addictologie, la Fédération Addiction a actualisé les avancées constatées en matière de JRP, et ouvert de nouvelles formes de coopération. En juillet 2023, les expérimentations de la JRP s’étendent à des territoires ultramarins et le département de Mayotte qui a manifesté son intérêt a été retenu.

La délinquance, comme l’addiction à quelque produit que ce soit, sont des phénomènes multifactoriels qui nécessitent d’agir dans plusieurs directions à la fois et d’actionner simultanément plusieurs leviers. Il s’agit d’une collaboration resserrée entre acteurs de la justice et de la santé dans l’accompagnement des personnes sous main de justice cumulant des facteurs de risque de récidive en matière de troubles addictifs, et de leur proposer, sur la base du volontariat, un parcours thérapeutique adapté et individualisé.

On parle de juridictions spécialisées, où la coopération entre une pluralité d’acteurs est centrale, et où la personne concernée se voit aussi conférer un « rôle » d’acteur dans la procédure. Cela procède d’un dispositif intégratif qui soutient et renforce la participation de l’usager à travers un « guichet unique », et un travail en commun des parties prenantes qui se réunissent régulièrement avec le justiciable.

La JRP s’appuie sur des connaissances scientifiques qui attestent de son efficacité, il ne s’agit pas pour autant d’un protocole préexistant qu’il faut scrupuleusement respecter, mais d’un cadrage théorique « souple » qui permet une adaptation aux contextes territoriaux selon une méthodologie éprouvée qui respecte le secret professionnel.

Le processus complexe de l’addiction et la survenue de possibles re-consommations sont pris en compte. On aborde la reconsommation sous l’angle motivationnel et non à travers une requalification pénale. Re-consommation n’est pas systématiquement synonyme de fin de l’inscription de la personne dans le dispositif. Consommations et récidives deviennent alors deux notions distinctes. S’engage un processus de rétablissement personnel qui vise à (re) trouver un équilibre, à améliorer la qualité de vie, et à renforcer la capacité d’agir de la personne. Le croisement de la notion de rétablissement avec celle de réduction des risques et des dommages permet de réduire les risques sanitaires et sociaux de récidive liés aux consommations, tout en respectant les besoins de la personne.

La JRP se veut un travail de coopération dont le bénéfice pour la personne est attesté, et le coût/efficacité pour les services de la justice avéré. La sociologie de Mayotte est aujourd’hui une invitation à penser collectivement un dispositif qui soit innovant.

# Research article: CheckNow : évaluation du déploiement et de l'efficacité de l'analyse de drogues en France

**Published:** 2024-06-24

**Authors:** Marion AUBERT, Pierre CHAPPARD, Perrine ROUX

**Affiliations:** Aix Marseille Université, Inserm, IRD, SESSTIM (Sciences **é**conomiques et sociales de la santé et traitement de l’information m**édicale**), Institut des sciences de la santé publique (ISSPAM), Marseille, France; Association PsychoActif, Paris, France

La diversité du marché des drogues et son évolution rapide augmentent la probabilité d’incertitude sur le contenu des produits en circulation, ainsi que les conséquences de cette incertitude sur la santé publique. Intégrée à l’offre en réduction des risques (RdR), l’analyse de drogue est un outil de promotion de la santé qui permet aux personnes utilisatrices de drogues (PUD) de connaître la composition de leur échantillon grâce à différentes techniques, gratuitement et anonymement. L’analyse de drogues – par des techniques qualitatives – ou quantitatives est aujourd’hui proposée dans de nombreux pays, y compris en France : cela nous donne l’opportunité d’accompagner son déploiement à l’échelle nationale, y compris dans les territoires d’outre-mer, et d’en évaluer les bénéfices auprès des plusieurs populations de PUD et dans différents contextes.

## Objectifs

Le projet CheckNow, financé par l’Institut pour la recherche en santé publique (IRESP) et l’Institut national du cancer (INCa), vise à évaluer l’analyse de drogues en termes de : 1) réduction des événements indésirables liés à la consommation (surdoses, conséquences négatives, etc.); 2) amélioration des connaissances, des compétences et des capacités d’agir des PUD (autonomisation, sentiment d’auto-efficacité). Il vise aussi à accompagner le déploiement de l’analyse de drogues à travers une description des contextes et des publics touchés ainsi que l’évaluation de son impact sur les pratiques professionnelles.

## Méthodes

L’impact d’une intervention sur la santé publique dépend de cinq dimensions : sa portée, son efficacité, son adoption par les professionnels et professionnelles du champ, sa mise en œuvre et son maintien. La méthode utilisée s’appuie sur le modèle de la recherche sur la mise en œuvre et permet de rendre compte de cet impact. Cette étude est aussi une recherche communautaire et participative, où les personnes concernées sont impliquées à toutes les étapes. Le projet se compose de trois phases :

- une phase exploratoire, *via* une enquête de type Delphi^2^2Consultation d’experts sur un sujet précis afin d’étudier le niveau de consensus. menée auprès d’experts et d’expertes de l’analyse de drogues pour préciser l’intervention à évaluer et renforcer les conditions de son évaluation;
- une phase d’évaluation quantitative, basée sur une étude longitudinale auprès de 1 000 PUD suivies pendant 12 mois avec 3 questionnaires, à l’inclusion, à 6 mois et 12 mois. Les analyses statistiques compareront un groupe de PUD ayant eu recours à l’analyse de drogues et un groupe n’ayant pas utilisé cet outil;
- une phase d’étude qualitative des perceptions et de l’acceptabilité menée, d’une part auprès des professionnels et professionnelles de la réduction des risques avec 3 groupes focaux et, d’autre part, auprès des PUD grâce à une étude qualitative de 30 entretiens semi-directifs.

## Retombées

Parallèlement à la description de l’impact de l’analyse de drogues sur les événements indésirables et les connaissances et compétences des PUD, ce projet permettra de comprendre les bénéfices de cette analyse auprès des professionnels et professionnelles de la réduction des risques ainsi que d’aider à la définition des modalités de sa mise en place.

# Research article: Maladies infectieuses émergentes : l'approche du clinicien

**Published:** 2024-06-24

**Authors:** Karim ABDELMOUMEN

**Affiliations:** Unité des maladies infectieuses et tropicales, Centre hospitalier de Mayotte, Mamoudzou, Mayotte, France

Le concept de maladies infectieuses émergentes (MIE) est né durant la 2^ème^ moitié du XXe siècle, dans l’ère-post-antibiotiques où les humains pensaient maîtriser les agents infectieux. Ce concept de MIE est défini comme suit : une entité clinique d’origine infectieuse nouvellement apparue ou identifiée, ou une maladie infectieuse connue dont l’incise augmente ou dont les caractéristiques se modifient. L’émergence préexiste à sa découverte et n’apparaît que pour celui qui la rencontre. L’anthropocène, défini comme l’ère moderne où l’humanité est la principale force de changement sur Terre à travers l’accroissement des sociétés humaines et la destruction des écosystèmes, crée un désordre planétaire inédit. L’anthropocène permet une variété infinie d’opportunités pour que des agents infectieux se transmettent aux humains et que de nouvelles maladies émergent dans un monde interconnecté où un virus peut littéralement faire le tour du monde en 24 h. Mayotte, île du sud-ouest de l’océan Indien est un département français, voisins des pays d’Afrique de l’Est, des Comores et de Madagascar. Mayotte a attiré l’attention des infectiologues ces dernières décennies par la survenue d’épidémies de dengue et aussi de chikungunya, Zika, leptospirose et fièvre de la vallée du Rift. Les échanges animaliers dans la région et la présence de vecteurs compétents à Mayotte font le lit rêvé des MIE et notamment des zoonoses. La proximité avec l’Afrique centrale et de l’Est force à rappeler un risque théorique de survenue de fièvre hémorragique (Ebola, Marburg). L’amélioration de nos outils diagnostiques (biologie moléculaire) a permis de diagnostiquer plusieurs cas de fièvre Q aiguë et chronique. Les praticiens mahorais sont habitués à la survenue d’épidémies d’arboviroses (dengue surtout) et de leptospirose, durant la saison des pluies. Plusieurs cas de mélioïdose ont été diagnostiqués à Mayotte. L’augmentation des résistances aux antibiotiques et notamment parmi les entérobactéries est un enjeu de santé publique à Mayotte avec une épidémiologie spécifique faisant aussi intervenir les bacilles Gram négatif non fermentant *(Pseudomonas aeruginosa* et *Acinetobacter baumanii).* La santé de l’environnement et des animaux doit être pensée au même niveau que la santé humaine. Mayotte peut ainsi devenir l’avantgarde de la révolution écologique et environnementale.

# Research article: Moustiques et risques vectoriels à Mayotte : état des lieux et travaux de recherche réalisés dans le cadre du partenariat *One Health* océan Indien

**Published:** 2024-06-24

**Authors:** Thierry BALDET, Ambdoul-Bar IDAROUSSI A, Diego AYALA, Renaud MARTI, Mathieu CASTETS, Jérémy BOUYER, Annelise TRAN

**Affiliations:** UMR ASTRE, Cirad/Cyroi, Sainte-Clotilde, La Réunion, France; Service de lutte antivectorielle, ARS Mayotte, Mayotte, France; Unité d’entomologie médicale, Institut Pasteur de Madagascar, Antananarivo, Madagascar.; MIVEGEC (Maladies Infectieuses et Vecteurs : Écologie, Génétique, Évolution et Contrôle), Univ. Montpellier, CNRS, IRD, Montpellier, France; UMR TETIS, INRAE, Montpellier, France; UMR TETIS, CIRAD, Saint-Pierre, La Réunion, France; UMR TETIS, CIRAD, Montpellier, France

Mayotte fait face à de nombreux défis sanitaires dont les maladies transmises par les moustiques. L’ile compte 45 espèces de moustiques avec une diversité notable, mélange de faune malgache et africaine incluant 4 espèces endémiques dont *Aedes pia.* Les espèces abondantes et agressives pour l’homme comportent *Aedes aegypti* et *Ae. albopictus* à activité diurne. Ces deux espèces sont des vecteurs majeurs des virus de la dengue en recrudescence depuis 10 ans et du chikungunya avec l’émergence majeure de 2005-2006. *Aedes albopictus,* d’installation récente dans l’île (2001), tend à supplanter *Ae. aegypti* en zone urbaine.

Citons aussi *Eretmapodites quinquevittatus,* espèce diurne abondante en forêt et vectrice potentielle du virus de la fièvre de la vallée du Rift ayant frappé Mayotte en 2007 et 2019. D’autres espèces nocturnes prolifèrent telles que *Culex quinquefasciatus,* nuisante et vectrice potentielle du virus West Nile circulant dans l’océan Indien. Dans le nord de l’île, *Anopheles gambiae* et *An. funestus* sont présents.

Grâce aux efforts de prévention et de lutte, le paludisme a fortement régressé dans l’île avec aucun cas autochtone depuis 2020. Le statut d’élimination du paludisme à Mayotte doit être consolidé face aux nombreux défis : maintien de populations résiduelles d’anophèles vecteurs, réintroduction régulière de parasites par des sujets humains infectés en provenance de zones impaludées dans un contexte où les problèmes d’habitat spontané et d’assainissement sont exacerbés par une démographie galopante dans l’île. En outre, un nouveau moustique envahissant menace l’océan Indien : *An. stephensi,* vecteur urbain du paludisme en Inde et maintenant en Afrique. Son installation dans les territoires insulaires de la région encore endémiques (Union des Comores, Madagascar), libérés du paludisme (Réunion, Maurice, Mayotte) ou ne l’ayant jamais connu (Seychelles) constitue une préoccupation majeure. Dans le même temps, la situation hyperendémique de la dengue en Asie du Sud-Est fait craindre l’introduction de nouveaux sérotypes/génotypes viraux comme la dengue 3 qui sévit actuellement au Bangladesh. Même si l’homologation récente de vaccins contre la dengue (Qdenga®) constitue une avancée significative, la surveillance entomologique et la lutte antivectorielle demeurent indispensables pour contrôler cette arbovirose majeure.

Face à ces risques vectoriels, le réseau régional de recherche et d’enseignement en partenariat avec *One Health* océan Indien www. onehealth-oi.org déploie trois axes d’activités : i) cartographie & modélisation, ii) surveillance & diagnostics et ii) prévention & contrôle. À Mayotte, l’outil de prédiction des dynamiques de population *dAedes* « ARBOCARTO » est utilisé par l’ARS de Mayotte pour optimiser la surveillance et la lutte^3^3https://www.mayotte.ars.sante.fr/la-carte-du-risque-vectoriel-de-lars-mayotte.

La surveillance aux points d’entrée est renforcée en tenant compte du risque invasif posé par *An. stephensi* et en incluant des outils innovants. Des travaux visent à caractériser le niveau et les mécanismes de résistance aux insecticides des vecteurs cibles. Ces activités dédiées au risque vectoriel sont déployées dans l’océan Indien suivant une approche *One Health* intégrative, pluridisciplinaire et associant les différentes parties prenantes. Elles bénéficient de l’appui de nombreux bailleurs et font écho aux besoins exprimés par les partenariats français et européen dans la région.

# Research article: Le béribéri à Mayotte

**Published:** 2024-06-24

**Authors:** Renaud BLONDÉ

**Affiliations:** Service de réanimation pédiatrique – Centre hospitalier universitaire (CHU) de La Réunion – Saint-Denis.; Service de réanimation médicale – Centre hospitalier de Mayotte, Mamoudzou, Mayotte, France

Cette maladie fréquente à Mayotte est due à une carence en vitamine B1 ou thiamine. Une grande partie de l’alimentation mahoraise comprend des mabawas (ailes de poulet) et du riz, aliments connus pour avoir une très faible teneur en thiamine. Les aliments riches en thiamine comme le porc ou la levure de bière sont exclus, la population étant en grande majorité de confession musulmane. Ainsi, à Mayotte, du fait de l’alimentation, il existe un terrain prédisposant à cette maladie carencielle.

La thiamine est indispensable au cycle de Krebs. Une fois ingérée, la thiamine est rapidement phosphorylée en pyrophosphate de thiamine (TPP), cofacteur d’un grand nombre de systèmes enzymatiques, dont la pyruvate déshydrogénase.

En l’absence de TPP, le pyruvate issu de la glycolyse ne peut plus emprunter la voie du cycle de Krebs. Le corps s’adapte et le pyruvate est réduit en lactate dans les muscles en utilisant la voie anaérobie.

Les deux populations les plus touchées à Mayotte par cette maladie sont les femmes enceintes (du fait de l’augmentation des besoins en thiamine) et le nourrisson allaité sans supplémentation vitaminique. Les jeunes mères ont souvent un régime quasi-exclusif à base de *oubou* (eau de riz, dont la teneur en thiamine est nulle), qui selon la culture mahoraise, est censé favoriser la lactation. Chez l’adulte, le tableau clinique peut-être neurologique avec une polynévrite sensitivomotrice, connue sous le nom de *lalavi* en shimoré, ou cardiaque avec une insuffisance cardiaque aiguë.

Chez le nourrisson, le motif de consultation est une détresse respiratoire, qui peut faire penser initialement à une bronchiolite, surtout en période épidémique. L’examen clinique retrouvant fréquemment une dyspnée de Kussmaul, une turgescence des jugulaires et un reflux hépatojugulaire doit faire redresser le diagnostic. Deux examens complémentaires disponibles rapidement sont alors à réaliser permettant de conforter le diagnostic : une gazométrie qui objective une acidose lactique souvent sévère et une échographie cardiaque qui montre une dilatation des cavités droites avec une hypertension artérielle pulmonaire.

Un dosage de thiamine est alors effectué, permettant le diagnostic de certitude *a posteriori.*

Le traitement consiste en une injection de thiamine de 100 mg en intraveineuse.

L’évolution est rapidement favorable avec une disparition de l’hyperlactatémie dans les heures suivantes, une amélioration de la détresse respiratoire et une normalisation de l’échographie cardiaque dans les 24 heures. La recharge vitaminique intraveineuse doit être poursuivie pendant une semaine avant de faire un relais par voie orale pendant un mois*.*

# Research article: Tétanos à Mayotte

**Published:** 2024-06-24

**Authors:** Renaud BLONDÉ

**Affiliations:** Service de Rréanimation pédiatrique – Centre hospitalier universitaire (CHU) de La Réunion – Saint-Denis.; Service de réanimation médicale – Centre hospitalier de Mayotte, Mamoudzou, Mayotte, France

Le tétanos est une maladie encore présente à Mayotte du fait de la difficulté à réaliser une couverture vaccinale suffisante, surtout dans la population immigrée. Depuis 2015, neuf cas ont été décrits : cinq chez des enfants, quatre chez des adultes avec une prépondérance masculine. La létalité a été nulle, grâce aux moyens modernes de réanimation disponibles sur cette île. Le tétanos est une toxi-infection à température normale due à un bacille Gram positif, *Clostridium tetani.* Il existe sous deux formes, la forme sporulée et la forme végétative. La forme sporulée, présente dans le milieu extérieur, est capable de résister à l’ébullition, aux désinfectants et peut survivre des années. Une fois introduite dans l’organisme, par le biais d’une plaie, elle perd sa forme sporulée et prend sa forme végétative. Elle va se multiplier en milieu anaérobie et sécréter deux toxines : la tétanolysine et la tétanospasmine. C’est cette dernière qui va être responsable de la symptomatologie de la maladie.

Elle est internalisée au niveau de la jonction neuro-musculaire, transportée dans l’axone de manière rétrograde jusqu’à la partie proximale du motoneurone et empêche la libération des neurotransmetteurs GABA et glycine, qui sont les neurotransmetteurs des interneurones inhibiteurs. La voie inhibitrice est ainsi interrompue, entraînant une activité non régulée du système nerveux moteur avec une hyperactivité des motoneurones, responsables de spasmes musculaires généralisés.

L’incubation est comprise entre 6 et 15 jours. Le premier symptôme à apparaître est le trismus, puis progressivement tous les muscles vont être atteints (période d’état) : les muscles rachidiens provoquant l’opisthotonos (dessiné par Charles Bellen en 1809), les muscles laryngés pouvant aller jusqu’à une dysphagie, les muscles respiratoires faisant craindre un blocage thoracique et les muscles abdominaux avec un ventre de bois. Le délai entre le premier symptôme et la période d’état est appelée « phase d’invasion », souvent comprise entre un et cinq jours. Plus elle est courte, plus le pronostic est réservé. Durant la période d’état, qui va durer entre troiq et cinq semaines, le patient est conscient et apyrétique. Durant la deuxième semaine, un syndrome dysautonomique peut se voir, correspondant à l’hyperactivité du système sympathique par blocage des neurones inhibiteurs*.* Le diagnostic est clinique.

Le traitement comprend trois volets : le traitement spécifique, le traitement symptomatique et le traitement des complications d’un séjour prolongé en réanimation.

Le traitement spécifique consiste à l’immunothérapie par voie IM (250 à 500 UI d’immunoglobuline tétanique), l’immunothérapie intrathécale, l’antibiothérapie par métronidazole pendant 7 jours (7,5 mg/kg/6h chez l’enfant et 500 mg/6 h chez l’adulte), la vaccination et le traitement de la porte d’entrée, quand elle est retrouvée*.*

Les benzodiazépines sont le traitement de choix des contractures musculaires avec des posologies qui vont être rapidement croissantes jusqu’à intuber le patient dans 75 % des cas. Après l’intubation, la curarisation est souvent utilisée. Des traitements antalgiques morphiniques sont indispensables. Il faut par ailleurs limiter les stimuli (bruits, soins, lumière), qui peuvent exacerber les contractures musculaires*).*

Le traitement des complications d’un séjour prolongé en réanimation consiste en de la ventilation artificielle, une nutrition entérale et souvent parentérale, le recours à un matelas anti-escarres, une anticoagulation préventive et le traitement souvent nécessaire des infections nosocomiales.

# Research article: Lutte contre l'antibiorésistance : définitions et grands principes du bon usage de l'antibiothérapie

**Published:** 2024-06-24

**Authors:** Bernard CASTAN, Mario BITAR, Carine BELZUNCE, Jean Benoit ZABBE, Alexandrine VIDAL, Claire AGUILAR

**Affiliations:** Service de maladies infectieuses et tropicales, Centre hospitalier de Périgueux.

Le développement de la résistance bactérienne aux antibiotiques est un problème mondial de santé publique depuis plusieurs années. Les leviers pour lutter contre ce phénomène sont multiples et la politique à mettre en œuvre s’inscrit dans une conception globale, résumée désormais sous la terminologie « Une seule santé ».

Le bon usage de l’antibiothérapie s’articule autour de la création d’indicateurs de surveillance de l’écologie bactérienne locale, d’un plan de formation des prescripteurs et d’information du grand public, du déploiement d’un dispositif d’aide au diagnostic et au traitement des infections. Ces politiques doivent intégrer des actions d’évaluation avec une programmation d’audits de bonne pratique et la création, la diffusion et la mise à jour d’un guide de prescription des anti-infectieux ou d’outils d’aide à la prescription sous la forme d’algorithmes décisionnels. Les modalités de prescription et de délivrance doivent être clairement définies en interne dans les établissements de santé en identifiant notamment une liste d’antibiotiques critiques. De telles listes sont élaborées au niveau international, tel que le projet AWARE de l’Organisation mondiale de la santé ou plus récemment la liste des antibiotiques critiques pour les établissements de santé et la médecine de ville élaborée par la Société de pathologie infectieuse de langue française (SPILF) en 2023 sur saisine de la Direction générale de la santé.

Ces programmes doivent être élaborés de façon collégiale par des équipes multidisciplinaires et faire l’objet d’une réévaluation régulière avec une présentation des résultats obtenus auprès des équipes de soins.

Depuis 2022, un maillage du territoire national se met progressivement en place autour des Centres régionaux en antibiothérapie (CRATB) et des équipes multidisciplinaires en antibiothérapie (EMA), mais ce modèle d’offre de soins doit être adapté au cas particulier de Mayotte.

# Research article: Épidémiologie basée sur les eaux usées en Guyane

**Published:** 2024-06-24

**Authors:** Marine COMBE

**Affiliations:** Institut de recherche pour le développement (IRD), UMR; ISEM, Montpellier, France

L’étude des eaux usées est un outil sentinelle permettant d’identifier la (co)-circulation de pathogènes au sein de la population, de détecter une émergence en cours et de suivre l’évolution de nouveaux variants. En Guyane, nous avons suivi la dynamique du SARS-CoV-2 et de ses variants dans les eaux usées et dans un sous-échantillon de tests nasopharyngés d’octobre 2020 à juillet 2021. Chaque mois, nous avons récolté 24 échantillons d’eaux usées (100 ml par site) au niveau de 24 points de refoulement (PR) répartis spatialement sur le territoire guyanais.

Les échantillons d’eaux usées ont été analysés par RT-qPCR afin de détecter la présence du virus et donc de suivre l’évolution de l’épidémie sur l’ensemble du territoire. Les résultats nous ont permis mois après mois d’informer les autorités locales telles que l’Agence régionale de Santé, la Préfecture et la collectivité territoriale sur le nombre et la localisation des sites de refoulement de eaux usées positifs au SARS-CoV-2.

Le suivi des eaux usées reflète la dynamique de l’épidémie au sein de la population, excepté au mois de mai 2021 quand le signal infectieux semble dilué par de fortes pluies. En parallèle, nous avons séquencé les échantillons d’eaux usées positifs ayant un CT-value < 38 en utilisant une méthodologie spécifiquement mise au point pour le COVID-19. Les ARN extraits ont été soumis à 98 amplifications PCR de 400 paires de bases en suivant le protocole ARTIC. Les librairies ont été préparées et soumises à un séquençage MinION (Oxford Nanopore). En parallèle, nous collaborions avec les hôpitaux de Guyane et l’Institut Pasteur sur des prélèvements nasopharyngés positifs pour le COVID, soumis au même protocole de séquençage. Nous avons séquencé 54 échantillons cliniques pour lesquels 100 % des échantillons ont pu être assignés pour des lignées, et 120 échantillons d’eaux usées pour lesquels seulement 20 d’entre eux ont été assignés. Dans les échantillons cliniques, nous avons observé une plus forte diversité des lignées du SARS-Cov-2, en lien avec le plus grand nombre d’échantillons positifs.

L’analyse des eaux usées a permis d’identifier les lignées majoritaires dans les deux types de matrices, ainsi que les variants brésiliens d’intérêt comme souligné par l’OMS. L’analyse en parallèle des eaux usées et des cas cliniques permet d’obtenir une bonne représentation de l’ensemble des variants circulants sur le territoire chez les porteurs symptomatiques et asymptomatiques.

Nous avons voulu connaître l’origine de la lignée guyanaise du SARS-CoV-2. Notre hypothèse de travail était que, du fait de forts effets frontaliers avec le Brésil et le Suriname, cette lignée guyanaise prend son origine dans les pays frontaliers. Nous avons réfuté cette hypothèse, constatant que cette lignée guyanaise avait son origine au sein d’une lignée européenne.

La présence et la diversité génétique d’autres pathogènes tels que les mycobactéries, les leptospires, la fièvre Q, les nématodes ont également été analysés à partir de ces prélèvements d’eaux usées. Ces études soulignent le potentiel de suivi épidémiologique que représentent les eaux usées, notamment en zone tropicale.

# Research article: Comparaison des cas humains de leptospirose entre Cayenne, Mayotte et La Réunion de 2018 à 2021

**Published:** 2024-06-24

**Authors:** Anissa DESMOULIN, Olivier MAILLARD, Louis COLLET, Renaud BLONDÉ, Jeanne Arline RAJAONA-RIVELO, Paul Le TURNIER, Marie Christine JAFFAR-BANdJEE, Anne Ovize, Pablo TORTOSA, Loïc EPELBOIN, Loïc RAFFRAY

**Affiliations:** 1 Unité des maladies infectieuses et tropicales, Centre hospitalier de Cayenne, 97300, Guyane, France; 2 Centre hospitalier universitaire de La Réunion, Saint Denis, 97400, La Réunion, France; 3 Centre hospitalier de Mayotte, Mamoudzou, 97600, Mayotte, France; 4 Centre d’investigation clinique, INSERM CIC 1410, CHU Sud Réunion, Saint Pierre, 97410, La Réunion, France; 5 Université de La Réunion, CNRS 9192, INSERM U1187, IRD 249, URM PIMIT, CHU de La Réunion, Uni-té mixte processus infectieux en milieu insulaire tropical (PIMIT), Plateforme technologique CYROI, Sainte-Clotilde, 97400, La Réunion, France; 6 Département de recherche, innovation et santé publique (DRISP), Centre hospitalier de Cayenne, 97300, Guyane, France; 7 Laboratoire Eurofins Biomnis, Lyon, 69007, France

## Introduction

La leptospirose est une zoonose causée par les bactéries du genre *Leptospira.* Peu de données existent évaluant la présentation clinique et la sévérité des cas humains. La Guyane, Mayotte et La Réunion sont trois départements d’outre-mer dont les incidences sont parmi les plus élevées de France. L’objectif de ce travail était de comparer la sévérité des cas humains de leptospirose de ces trois départements français.

## Méthodes

Il s’agit d’une étude rétrospective multicentrique incluant tous les patients âgés de plus de 18 ans avec une leptospirose prouvée par PCR sur la période de janvier 2018 à décembre 2021 au Centre hospitalier de Cayenne et de janvier 2018 à mai 2020 à Mayotte et à La Réunion. Les données démographiques, cliniques et biologiques ont été recueillies sur ces différentes périodes. Le critère de jugement principal était la sévérité définie par le recours à la ventilation mécanique, à l’épuration extra-rénale, aux amines vasoactives et/ou le décès.

## Résultats

Au total 560 patients ont été inclus (222 à Mayotte, 268 à La Réunion et 70 à Cayenne). Les patients étaient significativement plus jeunes à Mayotte (35 ans +/- 12) qu’à La Réunion et qu’à Cayenne (48 +/- 16 et 42 ans +/- 15 respectivement; p<0,001). Les cas étaient majoritairement des hommes dans les trois départements. Parmi les déclarations d’exposition, un contact avec des rats au domicile était plus fréquemment rapporté à Mayotte (57,5 %, 23/40) et à Cayenne (76,1 %, 35/46). À La Réunion, parmi les déclarants la voie d’exposition la plus fréquente était lors des activités professionnelles (44,6 %, 86/193). Il existait moins de formes graves à Mayotte (8,1 %, n=18), qu’à La Réunion (24,3 %, n=65) ou qu’à Cayenne (24,2 %, n=17; p<0,001). Parmi les patients hospitalisés, le recours à l’épuration extra-rénale était le plus fréquent à Mayotte (8,8 %, 9/102) et à La Réunion (22,3 %, 48/215), alors qu’il s’agissait du recours à la ventilation mécanique à Cayenne (14,5 %, 9/62). Les létalités globales observées étaient respectivement de 0,9 % (n=2), 1,5 % (n=4) et de 2,9 % (n=2).

## Discussion et conclusion

La leptospirose reste un problème majeur de santé publique, notamment dans les départements de la Guyane, de Mayotte et de La Réunion. Cependant, des différences peuvent être observées entre ces trois territoires, tant au niveau de la population qu’elle touche, qu’aux facteurs d’expositions et qu’à sa sévérité. Les cas de leptospirose en Guyane et à La Réunion nécessitent plus souvent un recours aux traitements de suppléances qu’à Mayotte. La létalité observée était comparable dans ces trois départements. Des études explorant l’implication majoritaire de certaines espèces dans la survenue des formes graves permettraient une meilleure orientation des stratégies de santé publique.

# Research article: AFROSCREEN : renforcement des capacités de séquençage génomique pour la surveillance des pathogènes émergents en Afrique

**Published:** 2024-06-24

**Authors:** Eric D’ORTENZIO

**Affiliations:** pour le consortium AFROSCREEN ANRS Maladies infectieuses émergentes (Inserm), France

La surveillance génomique est reconnue comme un outil de santé publique incontournable pour une meilleure compréhension des pathogènes, de leur évolution, de leur circulation et pour le développement de tests diagnostiques, de vaccins et de traitements. L’Organisation mondiale de la santé (OMS) plaide pour un accès aux avantages de la génomique de manière efficace, éthique et équitable. C’est dans ce contexte que s’inscrit le projet AFROSCREEN.

AFrOsCREEN est un projet multi-institutionnel qui vise à renforcer les capacités de séquençage et de surveillance génomique en Afrique subsaharienne afin d’alerter les autorités et la communauté internationale et de produire des données pour accompagner les décideurs dans la définition des priorités de santé publique.

Avec 25 partenaires dans 13 pays d’Afrique (Bénin, Burkina Faso, Cameroun, République centrafricaine, République démocratique du Congo, Ghana, Madagascar, Mali, Niger, République de Côte d’Ivoire, République de Guinée, Sénégal et Togo), le projet est coordonné par l’ANRS Maladies infectieuses émergentes (MIE) dans le cadre d’un consortium réunissant : l’ANRS MIE (Inserm), l’Institut Pasteur et l’Institut de recherche et développement (IRD), en coordination avec l’Africa CDC et l’OMS-AFRO. Il est financé par l’Agence française de développement à hauteur de 10 millions d’euros. Depuis 2021, AFROSCREEN a fourni du matériel et des réactifs aux laboratoires et a organisé 32 formations pour les équipes laboratoire, 12 pour les équipes d’épidémiologistes, et 6 webinaires.

La mise en place de la PCR de dépistage des mutations du SRAS-CoV-2 a permis l’identification du variant Omicron dans plusieurs pays. AFROSCREEN a fourni des séquenceurs à sept laboratoires et a contribué à l’augmentation du nombre moyen de séquences déposées sur GISAID *(Global Initiative on Sharing Avian Influenza Data)* par les pays (48 séquences/mois/pays ont été déposées en 2021,79 en 2022 et 28 en 2023, nonobstant la diminution progressive du nombre de cas de COVID-19).

En outre, AFROSCREEN a contribué à la mise en place et/ou au renforcement de 77 sites de surveillance sentinelle et à l’intégration de la surveillance SRAS-CoV-2 aux réseaux « grippe » existants.

Le projet a commencé à fournir les réactifs pour le diagnostic et la caractérisation des virus prioritaires dans les différents pays (Ebola, Marburg, monkeypox, rougeole, dengue, fièvre de la Vallée du Rift …) laboratoires du réseau sont en mesure aussi de s’impliquer dans les investigations animales et humaines, afin de faire des analyses de séquençage de pathogènes infectieux à haut potentiel épidémique dans une approche *One Health.*

Les principaux défis rencontrés jusqu’à présent concernent les délais d’approvisionnement en matériel et en réactifs, en raison de la faible priorité accordée aux pays africains par les fournisseurs, de la complexité et de la lenteur des procédures administratives, et de l’impact des relations diplomatiques entre les pays.

AFROSCREEN facilite la détection, la caractérisation et la surveillance génomique des agents pathogènes pour la préparation aux futures épidémies dans les pays africains. Les plateformes consolidées permettront également des projets de recherche qui soutiendront la capacité scientifique, tout en répondant aux défis des futures pandémies.

# Research article: Actualités sur l'angiostrongylose nerveuse dans l'océan Indien

**Published:** 2024-06-24

**Authors:** Loïc EPELBOIN, Anissa DESMOULIN, Louis COLLET

**Affiliations:** 1 Unité de maladies infectieuses et tropicales et Centre d’investigation clinique Antilles Guyane (CIC), France; 2 Unité des maladies infectieuses et tropicales, Centre hospitalier de Cayenne, 97300, Guyane Française Inserm 1424, Centre hospitalier de Cayenne Andrée Rosemon, Cayenne, Guyane, France; 3 Laboratoire de biologie médicale, Centre hospitalier de Mayotte, Mamoudzou, Mayotte, France

L’angiostrogylose nerveuse est une maladie parasitaire rare liée à un helminthe nématode *Angiostrongylus cantonensis.* C’est une zoonose responsable occasionnellement chez l’être humain d’une atteinte neurologique à type de méningite ou méningo-encéphalite à éosinophiles. Son hôte définitif est le rat, les hôtes intermédiaires sont des gastéropodes de type limaces ou escargots (en particulier l’escargot géant africain, *Achatina fulica,* une espèce envahissante partout dans le monde), et ses hôtes paraténiques des crustacés, grenouilles, lézards ou planaires. L’homme se contamine en ingérant, souvent accidentellement, de la bave d’escargot ou en consommant des crustacés infectés insuffisamment cuits. La maladie se traduit par des signes digestifs et neurologiques de gravité variable, des simples céphalées au coma mortel, associés à une hyperéosinophilie sanguine variable, et une cellularité dans le liquide cérébrospinal avec plus de 10 % d’éosinophiles. Le pronostic dépend de la forme clinique et de l’âge, avec des formes plus graves chez les enfants. Le diagnostic repose sur la sérologie, réalisée seulement à l’Institut tropical et de santé publique suisse de Bâle, ou surtout sur la PCR *(polymerase chain reaction)* dans le liquide cérébro-spinal (LCS), réalisée en France au Centre hospitalier de Mayotte (CHM) et à l’Institut Pasteur de Guadeloupe. Depuis les premiers cas humains décrits à Taïwan dans la première moitié du 20^e^ siècle, plusieurs épidémies ont été décrites en Chine et en Asie du Sud-Est et dans les îles du Pacifique (Hawaï, Nouvelle-Calédonie, Tahiti). Des cas ont été plus récemment décrits en Amérique latine et dans les Caraïbes, suite à la progression de l’aire de répartition de l’achatine.

Dans l’océan Indien, le parasite a été retrouvé dans des escargots et des rats, lors d’études réalisées au milieu des années 1960 à Madagascar et à l’île Maurice, puis dans la région de Tananarive chez des rats au début des années 1980, mais aucun cas humain n’y a jamais été rapporté dans la littérature. À La Réunion, des cas de méningites à éosinophiles très évocateurs ont été rapportés à la fin des années 1960 et au milieu des années 1970, puis des cas avérés ont été rapportés chez quatre nourrissons dans les années 1990, sans nouvelle occurrence connue jusqu’en 2023. À Mayotte, 6 cas de méningite à éosinophiles très sévères ont été rapportés dans les années 1990 chez des enfants de moins de 1 an, puis 3 nouveaux cas chez des 3 nourrissons de moins de 1 an et 3 chez des adultes entre 2000 et 2005. Entre 2007 et 2012, 14 cas ont été rapportés, dont 13 chez des enfants de moins de 2 ans, avec une létalité à 1 an de 36 % et des séquelles neurologiques chez 43 % des survivants. Près de la moitié rapportait un contact avec des achatines. Des explorations malacologiques dans les communes de résidence des enfants retrouvaient près de 10 % de positivité des achatines pour le parasite, avec des charges parasitaires importantes.

Entre janvier 2012 et septembre 2023, 21 prélèvements avec PCR *A. cantonensis* positives ont été identifiés au laboratoire du CHM. Après exclusion des doublons, on retrouvait 14 patients de Mayotte sur la période d’étude, dont 3 adultes et 11 enfants de moins de 12 ans parmi lesquels 9 de moins de 2 ans, et un sex-ratio autour de 1. On retrouvait également 3 cas autochtones diagnostiqués à La Réunion en 2023 chez des adultes de 52, 53 et 84 ans à Saint André, avec une évolution favorable.

Au total, si les données sont inexistantes à Madagascar et Maurice, il semble que la maladie soit toujours d’actualité dans les îles françaises de l’océan Indien, avec trois cas diagnostiqués à la Réunion en 2023 chez des adultes, et entre un et deux cas rapportés de façon régulière chaque année à Mayotte depuis 2007, majoritairement chez des enfants de moins de deux ans. Des investigations humaines et animales sont importantes à mettre en place, ainsi que des campagnes de sensibilisation de la population pour empêcher le contact des enfants avec les escargots.

# Research article: Fièvre Q en Afrique et dans l'océan Indien : une maladie tropicale négligée ?

**Published:** 2024-06-24

**Authors:** Loïc EPELBOIN

**Affiliations:** Unité de maladies infectieuses et tropicales et Centre d’investigation clinique Antilles Guyane (CIC) Inserm 1424, Centre hospitalier de Cayenne Andrée Rosemon, Cayenne, Guyane, France

## Introduction

La fièvre Q est une zoonose bactérienne ubiquitaire liée à *Coxiella burnetii.* Elle se transmet principalement par inhalation de produits de la mise bas issus des animaux de rente, bovins, ovins et caprins. Elle est à l’origine d’un tableau de fièvre associée de façon variable à une pneumopathie, une atteinte hépatique et à un syndrome inflammatoire biologique. L’évolution de la forme aiguë est généralement favorable même sans antibiotiques mais une évolution vers une forme chronique (endocardite ou infection vasculaire) peut survenir en particulier chez les patients immunodéprimés ou porteurs d’une valvulopathie.

Il existe de nombreuses régions du globe pour lesquelles aucune mention de coxiellose humaine ou animale n’est retrouvée dans la littérature médicale.

## Méthodologie

Une revue de la littérature a été réalisée avec comme mot clé *Coxiella burnetii* ou fièvre Q, et successivement tous les pays et territoires d’Afrique et du sud-ouest de l’océan Indien.

## Résultats

Les premières mentions de la fièvre Q remontent à une publication en 1955 dans plusieurs pays africains d’Afrique du Nord (Algérie, Maroc, Égypte, Libye et Tunisie), d’Afrique subsaharienne (République Démocratique du Congo, Cameroun, Kenya, Guinée Bissau et Säo Tome, Zimbabwe, Burkina Faso, République Centrafricaine, Soudan) et Afrique du Sud et de l’océan Indien (Madagascar).

Entre 1955 et 2023, en Afrique du Nord, on retrouve trois études de séroprévalence en Egypte et une en Algérie. Quelques cas d’endocardite sont rapportés en Algérie et en Tunisie. Aucune étude n’est retrouvée au Maroc, en Libye et au Soudan. En Afrique subsaharienne, une à deux études, soit de séroprévalence dans la population générale, soit de prévalence parmi des fièvres ou des pneumopathies, sont rapportées dans certains pays, à l’exception de deux pays d’Afrique de l’Est, le Kenya et la Tanzanie, où la littérature est un peu plus riche. On ne trouve aucune mention de cette pathologie dans plus de 20 pays d’Afrique subsaharienne. Dans les rares études réalisées, on observe une séroprévalence de 2 à 60 % selon les régions et les populations étudiées. La fièvre Q représente de 0,5 à 10 % des causes de fièvres aiguës dans certains pays, jusqu’à 9 % des séries de pneumopathies hospitalisées et de 1 à 3 % des endocardites. Les facteurs de risque retrouvés dans les grandes séries étaient principalement de vivre dans des zones rurales et d’avoir des activités en rapport avec le bétail. Des cas rapportés chez des voyageurs au retour d’Afrique font évoquer la présence de la bactérie, comme au Sud Soudan, en Afrique du Sud, ou au Malawi.

Dans l’océan Indien, une épidémie de pneumopathie documentée à *Coxiella burnetii* avait été rapportée en 1964 à Madagascar, puis un cas d’artérite attribuée à cette pathologie en 1977. Une étude publiée en 2018 ne trouvait aucune PCR à *C. burnetii* positive sur le sang de 1 020 patients fébriles entre 2011 et 2013. Aux Comores, une étude publiée en 1992 trouvait une séroprévalence de 5 % chez des donneurs de sang ou des volontaires sains et un cas de co-infection fièvre Q et paludisme a été rapporté en 2005 au retour des Comores. À Mayotte, seul un cas d’endocardite à *C. burnetii* chez une patiente anjouanaise porteuse d’une bioprothèse a été rapporté et l’analyse rétrospective sur 11 ans (2004-2015) de la sérologie *C. burnetii* du laboratoire de l’hôpital de Mayotte n’avait permis de retrouver aucun autre cas d’infection par la fièvre Q. En revanche, dans deux études de cohorte, l’une réalisée en 2011 sur 400 vaches et chèvres et l’autre en 2012 sur 98 moutons, la séroprévalence de la fièvre Q était respectivement de 10 % et de 5,1 %. Récemment, une PCR étudiant plus de 30 pathogènes a été réalisée sur 665 patients fébriles sur la période avril à septembre 2023 parmi lesquels 5 PCR *C. burnetii* étaient positives.

À La Réunion, la fièvre Q a été récemment démontrée comme endémique. Alors que seuls quelques cas d’endocardite avaient été décrits jusqu’au début des années 2010, des taux de séroprévalence élevés ont été récemment observés dans la population générale. Une étude de séroprévalence réalisée chez 1 112 femmes enceintes en 2014 a montré une séropositivité d’environ 20 % et d’environ 7 % pour 241 sérums datant de 2009. Une étude de séroprévalence chez les animaux de rente réalisée en 2011-2012 montrait une séroprévalence de 11,8 % des bovins, 1,4 % des ovins et 13,4 % des caprins (correspondant à 46 % des fermes de bovins, 50 % des fermes d’ovins, et 41 % des fermes de caprins).

Au total, la fièvre Q est probablement massivement sous-diagnostiquée en Afrique et dans l’océan Indien, et elle pourrait représenter une part non négligeable des endocardites, des pneumopathies aiguës communautaires et des fièvres aiguës. Il existence un besoin immense de recherche sur cette pathologie dans cette région du monde.

# Research article: Leptospiroses : Enjeux, actualités, et projets en Guyane

**Published:** 2024-06-24

**Authors:** Paul LE TURNIER

**Affiliations:** Unité de maladies infectieuses et tropicales, Centre hospitalier de Cayenne, Guyane, France

La leptospirose est une pathologie en recrudescence en Guyane depuis une dizaine d’années. Cela est probablement en lien avec une plus grande sensibilisation médicale et un meilleur recours aux outils diagnostics adaptés. De plus, si les conditions climatiques et les conditions de logement insalubres semblent propices à l’émergence de la leptospirose, de nombreux éléments demeurent encore incertains sur les cycles de transmission. Les données environnementales encore très parcellaires semblent en faveur d’une présence des leptospires en zone urbaine préférentiellement. Les données sur le réservoir animal sont limitées et anciennes : la dernière publication remonte à 1992 ! Au total, les sites de contamination pour les cas humains de leptospirose restent souvent méconnus : domicile, lieu de travail, zone d’activité de loisir extérieure. Des développements récents et à venir des ressources diagnostiques locales devraient permettre de mieux connaître les souches impliquées en pathologie humaine. La disponibilité de la PCR au sein de l’hôpital de Cayenne depuis fin 2022 pourrait faciliter l’identification précoce des cas et un meilleur recueil des expositions à risque. Plusieurs travaux portant sur l’étude des différents réservoirs animaux sauvages forestiers, urbains et périurbains, devraient apporter prochainement des réponses sur la prévalence de la leptospirose chez les animaux en contact rapproché avec l’homme (chiens, bétail, rongeurs). Ces travaux sont portés par différentes structures de recherche de Guyane et devraient être complémentaires.

Le rôle de l’orpaillage dans l’épidémiologie semblait significatif dans une étude de 2007 à 2014. De plus, une étude de séroprévalence a montré qu’un tiers des orpailleurs clandestins travaillant en Guyane s’étaient immunisés contre la leptospirose. Toutefois, l’augmentation des cas des dernières années semblerait plutôt concerner des individus provenant de zones d’habitat informels de zone urbaine et périurbaine, dont la croissance a été majeure au cours de la dernière décennie. Des enquêtes sont prévues pour mesurer l’impact de la leptospirose dans ces populations précaires. Elles permettront d’évaluer la part de personnes exposées à cette zoonose et d’identifier des facteurs associés. Des approches plus systématiques d’évaluation des expositions, notamment des zones géographiques fréquentées lors de la période d’incubation des cas de leptospirose, devraient apporter des éléments de réponse et faciliter la surveillance épidémiologique de cette zoonose, redevenue récemment à déclaration obligatoire. Enfin, la réponse opérationnelle autour des cas suite aux déclarations obligatoires reste à définir localement. Elle pourrait associer des investigations (au domicile, lieu de travail, etc.) incluant du dépistage de cas groupés et d’éventuelles analyses environnementales (sols, eaux, animaux à proximité) pour déterminer les cycles locaux de transmission. L’ensemble de ces travaux permettra à terme de faciliter les diagnostics, de mieux connaître les souches humaines, animales et environnementales ainsi que d’élaborer des stratégies d’information et de prévention vis-à-vis des populations identifiées comme les plus exposées.

# Research article: Le paludisme à Mayotte : historique et actualités

**Published:** 2024-06-24

**Authors:** Jean-François LEPÈRE

**Affiliations:** Centre médical de référence de Dzoumogné, Centre hospitalier de Mayotte, Mamoudzou, France

Mayotte, département français de l’océan Indien, est l’une des quatre îles de l’archipel des Comores. C’est une zone de transmission permanente du paludisme, quasi exclusivement à *Plasmodium falciparum* (95 % des cas). Les deux principaux vecteurs sont *Anopheles gambiae* et *An. funestus.*

Le premier programme de lutte instauré en 1976 associe une campagne de pulvérisations systématiques d’insecticide à effet rémanent (PIR) dans les habitations, une chimioprophylaxie de masse par chloroquine (CQ) chez les femmes enceintes et les enfants et l’administration d’un traitement présomptif par CQ à tout accès fébrile. Ce programme est rapidement efficace et à la fin des années 1980, le paludisme n’est plus considéré comme un problème de santé publique. Malheureusement la situation s’est détériorée. À la fin des années 1990, le nombre annuel de cas est en moyenne de 1 500 et l’incidence supérieure à 10 %o. En 2001 on enregistre le nombre record de 10 décès. Cette dégradation est principalement due à une résistance croissante à la CQ. Les autorités sanitaires instaurent alors plusieurs mesures de renforcement de la lutte antipaludique.

- Les moyens diagnostiques sont renforcés avec la mise à disposition de tests de diagnostic rapide dans toutes les structures de santé, ce qui met *de facto* fin à la pratique du traitement présomptif.
- La stratégie thérapeutique est modifiée. De 2002 à 2007 les accès simples sont traités par l’association sulfadoxine-pyriméthamine. À partir de 2007, on utilise une combinaison thérapeutique à base d’artémisinine : l’artéméther-luméfantrine.
- Un système de surveillance est mis en place avec notification systématique aux autorités de santé de tous les cas de paludisme diagnostiqués par les médecins et les biologistes de l’île.
- La lutte antivectorielle est réorganisée et renforcée. L’objectif est désormais de trois passages annuels dans chaque habitation de l’île pour des PIR. De 2012 à 2016, une campagne d’équipement de tous les foyers de l’île de moustiquaires à imprégnation durable a pris le relais des PIR.

De 2002 à 2022, 4 819 cas autochtones sont notifiés à Mayotte. L’incidence annuelle a régulièrement diminué de 10,3 %o en 2002 (1 649 cas) à moins de 0,01 %% en 2020 (2 cas). Depuis 2009, l’incidence des cas autochtones est inférieure à 1 %o. Le dernier cas acquis localement a été notifié en juillet 2020. L’arrêt de la transmission autochtone du paludisme semble donc être confirmé à Mayotte. L’île peut désormais être considérée comme une zone exempte de paludisme. Cependant, durant cette période 2002-2022, 1 932 cas importés seront observés. Ils proviennent principalement de l’Union des Comores (85 %), de Madagascar (8 %) et d’Afrique subsaharienne (7 %). Les vecteurs compétents étant toujours présents, le territoire reste sous la menace d’une réintroduction *via* ces cas importés.

Le programme de lutte contre le paludisme, en vigueur actuellement à Mayotte, doit rapidement être réorienté dans l’objectif de prévenir une réintroduction de la maladie. Au vu de l’environnement géographique de l’île, il devra comporter une politique volontariste de coopération régionale en matière de lutte contre la maladie.

# Research article: Actualités sur l'antibiorésistance à Mayotte et à La Réunion

**Published:** 2024-06-24

**Authors:** Guillaume MILTGEN

**Affiliations:** Laboratoire de bactériologie, Centre hospitalier universitaire (CHU) de La Réunion, site Félix Guyon, 97400, La Réunion, France; UMR Processus infectieux en milieu insulaire tropical (PIMIT), CNRS 9192, INSERM U1187, IRD 249, Université de La Réunion, 97490, Saint-Clotilde, La Réunion, France; Centre régional en antibiothérapie de La Réunion (CRAtb Réunion), Saint-Pierre, La Réunion, France

Les deux départements d’outre-mer de l’océan Indien (Mayotte et La Réunion) sont fortement impactés par le phénomène de l’antibiorésistance. Ils sont un carrefour migratoire en lien avec l’Afrique australe et le sous-continent Indien. De plus, du fait des prises en charge des patients qu’offrent le Centre hospitalier de Mayotte (CHM) et le Centre hospitalo-universitaire de La Réunion (CHUR), il existe de nombreux échanges de populations à visée sanitaire (EVASAN), renforçant les échanges entre Mayotte, La Réunion et les autres territoires du sud-ouest de l’océan Indien (SOOI).

La surveillance des résistances bactériennes aux antibiotiques s’organise progressivement à Mayotte, et des données sont déjà disponibles à La Réunion. On sait par exemple que 43,3 % des patients entrant en réanimation au CHM étaient porteurs d’entérobactéries BLSE (β-lactamases à spectre élargi) en 2019, contre 8,5 % en 2018 au CHUR.

D’autre part, Mayotte a connu l’émergence de bactéries dites hautement résistantes (BHR) telles que les entérobactéries productrices de carbapénèmase (EPC), les *Acinetobacter baumanii* résistants à l’imipénème (ABRI) ou les *Pseudomonas aeruginosa* multirésistants (PYO-MDR). Concernant les EPC, Mayotte est confrontée depuis 2015 à une épidémie d’*Enterobacter cloacae* IMI1 avec un clone majoritaire circulant de type ST820 *(sequence type)* probablement d’origine communautaire et responsable de plus de 60 patients colonisés ou infectés. Depuis 2015, on note également l’émergence de plusieurs clones de *Escherichia coli* NDM-5 (ST58, ST 167, ST405, ST410) ou OXA-181 (ST410), avec une diffusion à double composante à la fois clonale et plasmidique (plasmides épidémiques communs circulant entre les différents clones). Ces mêmes clones sont communs à La Réunion et à Mayotte. Concernant les ABRI, plusieurs clones circulent dans l’océan Indien porteurs des carbapénèmases OXA-23, OXA-24 ou NDM1. En 2019-2020, le CHUR a été confronté à une grave épidémie d’ABRI-Co (ABRI également résistants à la colistine) OXA23+NDM1 en réanimation polyvalente. Le patient index était d’origine comorienne, récemment hospitalisé au CHM puis transféré au CHUR. L’épidémie a compté au final 13 patients dont 8 infections et 6 décès (dont 4 imputables à la souche d’ABRICo). Les mesures d’hygiène drastiques mises en place ont permis l’éradication du clone (notamment au niveau environnemental) et la fin de l’épidémie. Concernant enfin les PYO-MDR, on observe depuis 2019 l’émergence polyclonale de *Ps. aeruginosa* producteur de la carbapénèmase NDM-1 dans la zone. Le clone majoritaire ST773 est actuellement responsable d’une épidémie impliquant plusieurs services au CHUR. Après analyses génomiques des souches en collaboration avec le Centre national de référence de la résistance aux antibiotiques, il s’avère que les souches de ST773 détectées au CHM et au CHUR sont identiques, avec un cas index originaire des Comores. Ce clone ST773 peut être considéré comme DTR et XDR, puisqu’il n’est actuellement plus sensible qu’à la colistine, la fosfomycine et dans certains cas à l’aztréonam.

Il est ainsi nécessaire de renforcer les collaborations entre Mayotte et La Réunion pour mieux appréhender à l’avenir ce phénomène de l’antibiorésistance dans des domaines comme la surveillance épidémiologique, l’hygiène hospitalière ou la recherche dans une approche *One Health*.

# Research article: L'Équipe mobile Santé Environnement de la Croix-Rouge française : un exemple d'approche communautaire pour prévenir les maladies à transmission hydrique, vectorielle et zoonotique en Guyane

**Published:** 2024-06-24

**Authors:** Margot OBERLIS

**Affiliations:** Croix Rouge française, Cayenne, Guyane, France

## Contexte

Territoire français ultra-marin situé au nord-est de l’Amérique du Sud, la Guyane compte 301 000 habitants début 2023. Avec plus de la moitié de la population vivant sous le seuil de pauvreté en 2018 et une croissance démographique élevée (+2,5 % par an), le nombre de personnes en situation de précarité et d’isolement ne cesse de croître. Concernées par l’existence de pathologies d’origine hydrique, ces dernières sont affectées par des épidémies récurrentes de maladies vectorielles (dengue, chikungunya, Zika) et des cas de leptospirose sont fréquemment signalés.

Cette situation sanitaire singulière peut s’expliquer par certaines spécificités parmi lesquelles figurent un taux élevé de non raccordement au réseau public d’eau potable (15 % *versus* 1 % pour la France hexagonale) ainsi qu’une proportion importante de personnes vivant dans des zones d’habitat spontané (41 % de bâti spontané en 2015 dont 24 % situé en secteur potentiellement insalubre).

En septembre 2023, 63 sites d’habitat spontané sont recensés pour les seules communes du littoral. Un peu plus de 20 000 personnes y vivent dans des conditions de précarité et d’insalubrité. Majoritairement isolée et en situation irrégulière, cette frange de la population a difficilement accès à l’information sanitaire et n’est pas desservie par les réseaux d’eau, d’assainissement ou d’électricité.

Le projet Équipe mobile santé environnement (EMSE) propose une approche en promotion de la santé qui vise l’autonomisation des habitants ainsi qu’une autonomie de fonctionnement au sein des quartiers.

## Méthode

L’EMSE, financée par l’Agence régionale de santé, les communes et la collectivité territoriale et portée par la Croix-Rouge française, est un projet de promotion de la santé basé sur une approche communautaire. Elle est composée d’une responsable et de trois médiateurs en santé. L’équipe intervient auprès des habitants des quartiers pour l’acquisition de connaissances sur les maladies liées à l’eau (zoonotiques, vectorielles) et sur les moyens de prévention. Ces actions se déclinent sous forme de maraudes d’informations et de sensibilisation pour l’approche individuelle, des animations collectives, la création de comités de quartiers constitués d’habitants volontaires et l’accompagnement à la mise en place d’actions pour l’approche collective. Elle encourage les initiatives communautaires dans une stratégie d’amélioration du cadre de vie et de réduction des risques sanitaires en lien avec des problématiques d’accès à l’eau potable, à l’hygiène et à l’assainissement (EHA).

## Résultats

Déployé depuis 10 mois, l’équipe a réalisé 88 interventions d’« aller-vers » permettant de sensibiliser près de 3 000 personnes sur l’hygiène des mains, le transport – le stockage et la consommation de l’eau potable, le traitement de l’eau à domicile, la dengue, la leptospirose et la gestion des déchets. Un peu plus de 550 personnes ont participé à des animations collectives sur les mêmes thématiques et trois actions collectives visant à prévenir l’apparition de gîtes larvaires et la prolifération de rongeurs ont mobilisé près de 120 personnes. L’implication des habitants a permis la constitution de huit comités de quartiers, responsables de l’organisation d’actions sanitaires et de la mobilisation des habitants.

## Conclusion

L’acquisition de nouvelles connaissances par la sensibilisation, un changement de comportement par l’accompagnement à la mise en place de nouvelles pratiques, la mobilisation sociale par la réalisation d’actions sanitaires collectives sont autant d’interventions rendues possibles par la médiation en santé. Cette approche communautaire en santé justifie la présence accrue de l’équipe EMSE au sein des quartiers ainsi qu’un déploiement sur le long terme, garant d’une autonomisation des personnes et, *in fine,* d’un impact sanitaire positif et pérenne.

# Research article: État des lieux sur les projets de recherche sur la leptospirose à La Réunion

**Published:** 2024-06-24

**Authors:** Loïc RAFFRAY, Olivier MAILLARD

**Affiliations:** 1 Université et Centre hospitalier universitaire (CHU) de La Réunion, UMR Processus infectieux en milieu insulaire tropical (PIMIT), CNRS 9192, INSERM U1187, IRD 24, Île de La Réunion, France; 2 Département de santé publique et Centre d’investigation clinique INSERM CIC 1410, CHU Réunion, Île de La Réunion, France

La leptospirose est une maladie zoonotique d’importance mondiale touchant plus d’un million de personnes par an avec une létalité de 6 % en moyenne. Les pays des zones tropicales sont plus touchés du fait de conditions favorables à la survie et au développement des bactéries *Leptospira* responsables de l’infection. En santé humaine, la maladie se présente le plus souvent sous la forme d’un syndrome dengue-like mais peut engendrer une défaillance multi-viscérale. Au sein de la zone océan Indien, tous les territoires ont pu être analysés pour identifier les mammifères infectés ou réservoirs de la maladie. Nous disposons de données de santé humaine pour les territoires de La Réunion, de Mayotte et des Seychelles principalement. Les efforts de recherche se poursuivent pour mieux préciser des questions sur les aspects en santé humaine ou animale, et les interactions avec l’environnement selon l’approche *One Health.* Nous nous proposons de faire un point d’étape sur les études cliniques menées au sein des centres hospitaliers, sans prétendre à une revue exhaustive de toutes les études ou acteurs de la recherche sur cette thématique. D’autres recherches en cours non promues par l’hôpital sont simplement citées : recherches au sein des unités UMR (PIMIT), études épidémiologiques de Santé publique France, études vétérinaires (CIRAD).

Depuis début 2021, des patients sont inclus dans la cohorte COLEPT (cohorte de patients hospitalisés atteints de leptospirose), avec un suivi d’un an pour explorer diverses thématiques (gravité clinique, microbiologie, épidémiologie, évolution notamment). Cette étude concerne les quatre centres hospitaliers publics de La réunion. Elle a atteint l’objectif de 165 patients avec leptospirose confirmée inclus depuis mai 2023. La fin de suivi est prévue pour mai 2024 avec des résultats escomptés courant 2025. En parallèle, des études ancillaires sont menées. L’étude IMMUNOLEPTO vise à analyser la réponse immunitaire (cellules sanguines, cytokines sériques) des patients en phase aiguë et en convalescence pour déceler d’éventuels déterminants immunitaires associés au phénotype et au pronostic. L’étude LEPTONIC initiée en 2022 a pour objectif de suivre les patients jusqu’à deux ans après l’infection pour évaluer la persistance de symptômes chroniques. Toutes ces études sont en cours et ont permis de fédérer cliniciens et acteurs de la recherche pour assurer un travail prospectif de qualité, constituer une banque d’échantillons biologiques à divers temps de l’infection. Les résultats attendus pourraient avoir un impact sur la prise en charge des patients et sur la prévention, mais pourraient aussi promouvoir d’autres études sur la thématique.

# Research article: L'épidémie de peste pulmonaire à Madagascar en 2017

**Published:** 2024-06-24

**Authors:** Rindra Vatosoa RANDREMANANA, Jean Marius RAKOTONDRAMANGA, Voahangy ANDRIANAIVOARIMANANA, Beza RAMASINDRAZANA, Fanjasoa RAKOTOMANANA, Feno Jacob RAKOTOARIMANANA, Laurence BARIL, Minoarisoa RAJERISON

**Affiliations:** Institut Pasteur de Madagascar, Antananarivo, Madagascar

La peste est une maladie endémique à Madagascar. Au niveau mondial, Madagascar reste le pays le plus affecté par la peste en déclarant plus de 50 % de tous les cas de peste humaine signalés. Environ 300 cas sont notifiés chaque année dans des foyers déjà connus. La peste est une maladie à déclaration obligatoire avec un circuit de transmission des données cliniques et biologiques au niveau du Laboratoire central de la peste de l’Institut Pasteur de Madagascar (IPM). Chaque échantillon biologique d’un cas suspect de peste transmis à l’IPM fait l’objet d’un test de diagnostic rapide, d’une mise en culture pour l’identification des souches de *Yersinia pestis* et de tests de sensibilité aux principaux antibiotiques, et de biologie moléculaire quantitative pour confirmation. En fonction des résultats biologiques, ces cas notifiés sont classés en cas confirmés, probables ou suspects.

Du mois d’août à novembre 2017, Madagascar a subi une épidémie de peste avec une prédominance de la forme pulmonaire et en dehors des foyers habituels. Environ 2 414 cas de peste ont été notifiés dont 77,8 % de peste pulmonaire, 16,4 % de peste bubonique, 5,8 % de formes non précisées et un cas de peste septicémique. Plus de la moitié des cas notifiés de peste (61,5 %) provenaient du Grand Antananarivo et 8,3 % de la ville de Toamasina. Le pic de notification a été observé au cours des deux premières semaines du mois d’octobre 2017 avec 400-450 cas notifiés de peste pulmonaire par semaine. La létalité globale pour les cas de peste pulmonaire et bubonique a été de l’ordre de 6 %, et de 25 % pour les cas confirmés. La proportion des cas notifiés chez les enfants de moins de cinq ans a été de 26,8 % et 18,6 % des formes pulmonaires ont été des cas confirmés ou probables. Cinquante souches, toutes sensibles aux principaux antibiotiques ont été isolées : 8 chez des cas de peste pulmonaire et 49 chez des cas de peste bubonique.

# Research article: Épidémiologie du VIH et des hépatites à Mayotte

**Published:** 2024-06-24

**Authors:** Karim ABDELMOUMEN

**Affiliations:** Unité des maladies infectieuses et tropicales, Centre hospitalier de Mayotte, Mamoudzou, Mayotte, France

Le VIH reste un enjeu de santé publique mondial notamment en Afrique subsaharienne où l’incidence y est globalement en baisse ces dernières années. L’île de Mayotte est le dernier territoire d’outre-mer à être départementalisé. Le 101^ème^ département français est le plus pauvre du territoire avec 77 % de la population qui vit sous le seuil de pauvreté et le plus jeune avec 50 % de la population qui a moins de 20 ans. Du fait de son histoire coloniale et de sa géographie, Mayotte est aussi une zone où se jouent plusieurs enjeux migratoires avec des flux entrants des pays alentours : Comores, Madagascar, mais aussi de la région des Grands Lacs en Afrique (Rwanda, Burundi, République Démocratique du Congo). L’épidémiologie du VIH à Mayotte est un reflet des enjeux sanitaires régionaux en océan Indien. Depuis 2019, une augmentation significative de l’incidence du VIH a été observée. La file active des PVVIH suivis à Mayotte est actuellement (septembre 2023) de 490 patients. Le nombre de nouveaux patients dépistés sur le territoire mahorais était en moyenne de 34 par an jusqu’en 2020. En 2022, 75 nouveaux patients ont été dépistés. Ce nombre a été atteint en seulement six mois en 2023. Non seulement nous observons cette augmentation d’incidence, mais la part de patients au stade sida augmente également. La part des patients nouvellement diagnostiqués à Madagascar a augmenté de façon spectaculaire, passant de 25,6 % en 2019 à 45,3 % en 2022. Ces trois dernières années ont aussi été marquées par une augmentation de l’immigration en provenance de la région des Grands Lacs en Afrique. En 2023, Mayotte connaît une flambée épidémique du VIH et devient le département avec la plus forte incidence de cette rétrovirose. La transmission est hétérosexuelle principalement. La prédominance de femmes au sein de la file active fait craindre une partie immergée de l’iceberg. Concernant les hépatites virales, Mayotte présente une prévalence de l’hépatite B de 3 % soit 10 fois plus qu’en France métropolitaine. La prévalence de l’hépatite C et de l’hépatite delta sont faibles. La morbi-mortalité liée au VHB n’est pas assez documentée à Mayotte. Il s’agit d’un enjeu majeur de santé publique dont les instances publiques doivent s’emparer. Rappelons qu’il existe un vaccin sûr, efficace et accessible pour l’hépatite B.

Des actions fortes de dépistage et de sensibilisation sont attendues sur le territoire. Un accueil digne et humain des migrants et migrantes est indispensable pour aider à juguler l’épidémie.

# Research article: Infections opportunistes des PVVIH à Mayotte

**Published:** 2024-06-24

**Authors:** Karim ABDELMOUMEN

**Affiliations:** Unité des maladies infectieuses et tropicales, Centre hospitalier de Mayotte, Mamoudzou, Mayotte, France

Nous avons vu dans une précédente présentation que l’épidémie de VIH à Mayotte est en pleine expansion, le département devenant en 2023 le 1^er^ département en termes de nouveaux cas pris en charge en France. L’incidence augmente mais la part de patients au stade sida augmente également. Malgré cela, le nombre de décès lui n’augmente pas. Parmi les personnes nouvellement diagnostiquées au stade sida, 20 % sont originaires de Mayotte, 41 % des Comores, 31 % de Madagascar et 35 % de la région des Grands Lacs en Afrique. Les infections opportunistes (IO) les plus fréquentes compliquant le VIH sont la pneumocystose pulmonaire, la tuberculose, les infections à cytomégalovirus (CMV), la candidose œsophagienne puis la toxoplasmose cérébrale.

En 2022, nous avons dénombré 34 hospitalisations pour complications d’une infection VIH : 1 ostéo-arthrite tuberculeuse, 3 tuberculoses pulmonaires, 4 tuberculoses ganglionnaires, 3 miliaires tuberculeuses, 1 tuberculose neuro-méningée, 2 méningoencéphalites à CMV, 1 kaposi digestif (7 séjours), 1 aspergillose pulmonaire, et 1 cryptococcose. Sur les 8 premiers mois de l’année 2023, nous avons dénombré 25 hospitalisations : 3 toxoplasmoses cérébrales, 2 cryptococcoses, 2 kaposi digestifs, 5 pneumocystoses dont 1 associée à une candidose œsophagienne et 1 associée à une tuberculose disséminée, 1 tuberculose pulmonaire, 1 tuberculose ganglionnaire et 1 ostéo-arthrite tuberculeuse.

Les IO sont une complication grave d’une infection VIH à un stade avancé. La morbimortalité et le coût associé sont importants. Une offre de dépistage large sur le territoire et un accueil digne des personnes migrantes cumulant des facteurs sociaux de vulnérabilité sont indispensables.

# Research article: Traitement et qualité de vie des personnes vivant avec le VIH en Nouvelle Aquitaine

**Published:** 2024-06-24

**Authors:** Diana BARGER, Victor HEMAR, Mojgan HESSAMFAR, Didier NEAU, Sophie FARBOS, Olivier LELEUX, Charles CAZANAVE, Nicolas ROUANES, Pierre DUFFAU, Estibaliz LAZARO, Patrick RiSPAL, François DABIS, Linda WITTKOP, Fabrice BONNET

**Affiliations:** 1 Université de Bordeaux, INSERM, BPH, U1219, F-33000 Bordeaux, France; 2 Centre universitaire hospitalier (CHU) de Bordeaux, Service de médecine interne et maladies infectieuses, Hôpital Saint André, F-33000 Bordeaux, France; 3 CHU de Bordeaux, COREVIH Nouvelle Aquitaine, INSERM, U1219, F-33000 Bordeaux, France; 4 CHU de Bordeaux, Service des maladies infectieuses et tropicales, INSERM, U1219, F-33000 Bordeaux, France; 5 Centre hospitalier de la Côte Basque, Service de maladies infectieuses, F-64000 Bayonne, France; 6 Université de Bordeaux, INSERM, Institut Bergonié, BPH, U1219, CIC-EC 1401, F-33000 Bordeaux, France; 7 Centre hospitalier de Périgueux, Service de médecine polyvalente, F-24019 Périgueux, France; 8 Université de Bordeaux, Département d’immunologie, CNRS, ImmunoConcEpT, UMR 5164, F-33000 Bordeaux, France; 9 CHU de Bordeaux, Service de médecine interne, F-33604 Pessac, France; 10 Centre hospitalier de Agen-Nerac, Service de médecine interne, F-47923 Agen, France; 11 INRIA, SISTEM Team, F-33400 Talence, France; 12 CHU de Bordeaux, Service d’information médicale, INSERM, Institut Bergonié, CIC-EC 1401, F-33000 Bordeaux, France

## Introduction

La Qualité de vie liée à la santé (QdVLS) se rapporte à la manière dont les patients perçoivent l’impact de la maladie et du traitement sur leur vie, y compris les aspects physiques, psychologiques et sociaux.

Bien que ce ne soit pas un sujet récent, le contexte du traitement universel et l’augmentation de l’espérance de vie des personnes vivant avec le VIH (PVVIH) modifient notre perspective sur la QdVLS.

## Méthodes

Dans le cadre de l’étude QuAliV, sous-étude d’une cohorte prospective hospitalière de PVVIH suivis pour le VIH-1 en Nouvelle-Aquitaine (ANRS CO3 AQUIVIHNA), nous avons évalué la QdVLS et d’autres résultats tels que le syndrome dépressif (SD). Entre 2018 et 2020, plus de 900 PVVIH ont participé en remplissant les questionnaires validés : WHOQOL-BREF pour la QdVLS et le PHQ-8 pour le SD.

## Résultats

Les scores médians de QdVLS (échelle de 0 à 100) étaient plus élevés pour la qualité de vie physique (69, IQR [56, 81]) et environnementale (69, IQR [56, 75]), et plus faibles pour la qualité de vie sociale (56, IQR [44, 69]) et psychologique (56, IQR [44, 69]). Les PVVIH présentant >3 comorbidités, déclarant ressentir de la stigmatisation, ou ayant des revenus <1 500€/mois avaient des scores de QdVLS médians ajustés plus bas par rapport aux groupes de référence. Dans une analyse comparant la prévalence du SD chez les hommes et les femmes vivant avec le VIH à celle de la population générale (données de l’enquête EHIS-ESPS), la prévalence brute du SD était de 22,5 % chez les femmes vivant avec le VIH (IC à 95 % : 17,1-27,9) et de 20,8 % chez les hommes vivant avec le VIH (17,8-23,8), tandis que la prévalence pondérée était de 23,4 % et 21,7 % respectivement. Le ratio de prévalence brute était 2,49 (1,923,22) et 4,20 (3,48-5,05) fois plus élevé chez les femmes et les hommes vivant avec le VIH, respectivement, par rapport à leurs homologues de la population générale, et 1,46 (1,091,95) et 2,45 (1,93-3,09) fois plus élevé après ajustement pour les facteurs non spécifiques au VIH. Ensuite, nous avons comparé la prévalence du SD chez les femmes et les hommes vivant avec le VIH en fonction de leur niveau de stigmatisation (aucune, modérée et sévère) par rapport à leurs homologues de la population générale. Après ajustement pour les facteurs non spécifiques au VIH, la prévalence du SD chez les femmes vivant avec le VIH classées comme « non stigmatisées » n’était plus supérieure à celle de la population générale. Cependant, elle restait deux fois plus élevée chez les hommes vivant avec le VIH dans la même catégorie par rapport à la population générale.

## Discussion/Conclusion

Les résultats de ces deux analyses soulignent la nécessité de s’attaquer aux facteurs structurels, en plus des facteurs indirectement liés au VIH (comme la stigmatisation et la multi-morbidité), afin de rendre la vie des PVVIH la plus harmonieuse possible sur les plans physique, mental et social.

# Research article: Prise en charge médicamenteuse des patients chroniques à Mayotte : spécificités, contexte et limites

**Published:** 2024-06-24

**Authors:** Makrem BEN REGUIGA

**Affiliations:** Pharmacien hospitalier, vice-président de la Commission médicale d’établissement (CME), Centre hospitalier de Mayotte, Mamoudzou, Mayotte

Le système de santé à Mayotte diffère des autres territoires français sur les modalités de prise en charge des patients défavorisés, assurés sociaux (AS) sans complémentaire santé (SCS) et patients non affiliés sociaux (NAS), en l’absence, sur le territoire, de l’application du droit à l’aide médicale d’État (AME) et à la complémentaire santé solidaire (anciennement couverture médicale universelle complémentaire). La prise en charge pharmacologique de ces patients reste indispensable dans un territoire à forte prévalence de pathologies chroniques : plus 12 % pour le diabète et plus de 38,4 % pour l’hypertension artérielle (HTA) chez une population où 55,9 % des personnes âgées de 15 ans et plus étaient en surpoids ou obèses en 2019. Les pathologies infectieuses ne sont pas en reste avec une progression constante des cas de VIH diagnostiqués, dont le nombre a plus que triplé en huit ans (416 en 2022 *vs* 132 en 2014).

Depuis les années 2000, et afin de proposer une solution pour la prise en charge de ces patients, un fonctionnement dérogatoire aux règles de droit commun a été mis en place avec l’appui des autorités sanitaires de l’époque : l’unique hôpital du territoire a dû installer cinq structures de dispensation à titre humanitaire et pour les indigents (DHI) dépendantes de la pharmacie à usage intérieur (PUI) du Centre hospitalier de Mayotte (CHM). Cette décision institutionnelle est le fondement d’un système de soins à deux vitesses où l’hôpital public doit porter une responsabilité dans la prise en charge thérapeutique d’une partie majeure de la filière ambulatoire de Mayotte. Ces structures pharmaceutiques ont vu ainsi leur activité augmenter avec nombre de patients NAS ou AS-SCS. Le nombre d’ordonnances traitées est passé de 4 000 à 9 000 entre 2005 et 2006, et à 257 000 en 2019, chiffre qui s’est stabilisé depuis.

Ce système a montré ses limites : initialement bouée de sauvetage pour le système de santé à Mayotte, les pharmacies DHI sont devenues une menace pour la prise en charge des patients; non outillées pour répondre aux besoins de cette filière ambulatoire, elles sont non conformes aux règles minimales de qualité et de sécurité des soins avec un exercice en conditions dégradées : absence de pharmaciens, fonctionnement sans responsabilité pharmaceutique, absence d’analyses pharmaceutiques, de logiciels d’aide à la dispensation et de dossier pharmaceutique, d’accompagnement et d’éducation pharmaceutique pour les patients souffrants de pathologies chroniques et un thésaurus limité à une centaine de molécules. Par ailleurs, ces structures ferment leurs portes à chaque aléa touchant le service public hospitalier (plans blancs, crises sociales, droits de retrait, crises sanitaires comme le COVID-19, sous-effectifs et absentéisme…), entraînant parfois des fermetures prolongées et privant les patients pendant des semaines de traitements. Ces patients décompensent et se retrouvent pris en charge dans les filières de soins critiques. Le poids pharmaco économique de ces activités est lourd : ainsi, pour le premier semestre 2023, les achats d’un médicament anti-VIH (bictégravir/tenofovirAF/emtricitabine ou Biktarvy®) ont représenté le premier poste de dépense avec plus d’un million d’euros et 7 % des montants des achats pharmaceutiques du CHM sans financement institutionnel fléché.

La prise en charge médicamenteuse des patients NAS et AS-SCS est aujourd’hui dans une impasse. Pourtant, il existe des alternatives, dans un contexte où l’offre pharmaceutique libérale à Mayotte est particulièrement dense, avec une démographie pharmaceutique qui ne cesse de croître et qui assure un maillage territorial étroit et de proximité pour les patients. Des solutions concrètes intermédiaires entre le système actuel et l’AME ont été proposées par les acteurs de santé libéraux et hospitaliers à Mayotte. Nous les avons présentées au cours du colloque. Néanmoins, ces solutions nécessitent une politique volontariste de la part des autorités sanitaires dans l’intérêt actuel et futur de la santé publique à Mayotte et celui des patients du territoire.

# Research article: Actualités VIH 2023

**Published:** 2024-06-24

**Authors:** Bernard CASTAN, Joseph KOFFI, Mario BITAR, Carine BELZUNCE, Jean Benoit ZABBE, Alexandrine VIDAL, Claire AGUILAR

**Affiliations:** Service de maladies infectieuses et tropicales, centre hospitalier de Périgueux, France

Les progrès thérapeutiques des dernières années permettent désormais d’obtenir une indétectabilité durable de la charge virale chez la plupart des personnes vivant avec le VIH (PVVIH) et traitées. Les actualités présentées en 2023 sont encore très riches et cette présentation se concentre sur quelques données exposées lors des congrès internationaux de la CROI (Conference on Retroviruses and Opportunistic Infections) et de l’IAS (International AIDS Society) 2023).

Le nombre de nouveaux diagnostics d’infection a diminué en France en 2020 et 2021 avec une baisse des diagnostics à un stade précoce. Mais ce résultat ne reflète pas la tendance inverse observée à Mayotte. Si les résultats des indicateurs se rapprochent progressivement des objectifs du plan de l’ONUSIDA, de grandes disparités persistent à travers le monde. Les principales causes de mortalité des personnes vivant avec le VIH (PVVIH) sont désormais majoritairement indépendantes de l’infection VIH rendant compte de l’efficacité des thérapeutiques et du vieillissement de la population.

Les résultats de l’essai Alliance semblent démontrer un avantage au bictegravir comparativement au dolutegravir sur le traitement de l’infection par le virus de l’hépatite B chez les patients coinfectés. Chez les PVVIH coinfectées par la tuberculose, le dolutégravir à dose standard semble aussi efficace que la double dose malgré l’interaction avec la rifampicine. Une étude taïwanaise confirme l’intérêt de la rifapentine dans des schémas thérapeutiques courts, en particulier dans le traitement des infections tuberculeuses latentes. Plusieurs études menées à terme soulignent l’intérêt croissant pour les thérapeutiques « *I ong acting* » par voie parentérale. L’essai SOLAR en simplification chez les patients prétraités sans échec préalable pour les classes des molécules concernées (inhibiteurs non nucléosidiques de la transcriptase inverse et inhibiteurs d’intégrase) ou dans le cadre de la PrEP (prophylaxie pré exposition) l’illustre. Dans ce cadre particulier, un syndrome rare a été décrit sous l’acronyme LEVI (Longacting Early Virology Inhibition syndrome), imposant une vigilance particulière pour ne pas méconnaître un diagnostic d’infection en cas d’échec de la PrEP. L’étude SOLAR3D permet de démontrer l’absence d’impact d’une mutation M184V en cas de *switch* vers une bithérapie DTG/3TC chez des patients indétectables avec une durée médiane d’inhibition virologique de 11,8 ans avant simplification thérapeutique. De nombreuses données concernent également le profil de tolérance et la pharmacocinétique des antirétroviraux au cours de la grossesse ou en cas de comorbidités.

C’est dans ce contexte que nous attendons avec impatience le prochain rapport de la Haute autorité de santé (HAS) sur la prise en charge des PVVIH.

# Research article: Comment atteindre les objectifs 3 × 95 de l'ONUSIDA dans le département singulier de Mayotte ?

**Published:** 2024-06-24

**Authors:** Victoire COTTEREAU

**Affiliations:** Géographe de la santé et des migrations internationales, Maitre de conférences au Centre universitaire de formation et de recherche (CUFR) de Mayotte, Laboratoire EspaceDev, Mayotte

Les données de l’ONUSIDA montrent qu’aujourd’hui, 29,8 millions des 39 millions de personnes vivant avec le VIH dans le monde reçoivent un traitement antirétroviral. Dans le cadre des Objectifs de développement durable (ODD), les objectifs 3 x 95 ont été pensés dans le but de mettre un terme à l’épidémie de sida à l’horizon 2030. Concrètement, cela signifie que :

- 95 % des personnes atteintes de VIH connaissent leur état sérologique;
- 95 % de ces personnes suivent un traitement antirétroviral vital;
- 95 % des personnes sous traitement ont une charge virale indétectable.

Dans le sud-est de l’Afrique, plusieurs pays ont déjà atteint ces objectifs. Face à ces ripostes au VIH couronnées de succès, pourquoi Mayotte, qui a longtemps été épargnée par l’épidémie, est aujourd’hui le territoire français avec la proportion de sérologies positives la plus élevée avec 3,5 pour 1 000 ? Le contexte économique et social de l’île est un facteur explicatif de ces données. Mayotte est marquée par une forte croissance démographique. La population est jeune, et un habitant sur deux est de nationalité étrangère. Les constructions en tôle sont nombreuses et 77 % de la population vit sous le seuil de pauvreté. La majorité des habitants n’est pas diplômée et souffre d’illettrisme ou d’analphabétisme. L’Aide médicale d’État (AME) n’y est pas appliquée et près de 30 % de la population ne dispose d’aucune couverture maladie. La précarité sociale de la majorité des habitants entraîne des inégalités d’accès et un fort taux de renoncement aux soins pour des raisons financières et/ou spatiales. Cette précarité est également un facteur d’amplification du phénomène de la prostitution.

La promotion de la santé sexuelle et la prévention ont véritablement pris forme ces dernières années, à l’instar de la campagne d’affichage menée par l’association Nariké M’Sada. Celle-ci met en scène de vrais couples qui font la promotion du dépistage, signe d’un réel progrès dans une société musulmane où le sujet de la sexualité est encore tabou. Par ailleurs, le système de soins est en hypertension et il n’existe que cinq lieux de dépistage permanents (hors PMI). Seul le Centre gratuit d’information, de dépistage et de diagnostic (CEGIDD) propose des dépistages du lundi au vendredi au sein d’un dispensaire à Mamoudzou qui est quotidiennement saturé. Pour les personnes non affiliées à la Sécurité sociale n’ayant pas les moyens financiers de s’y rendre et/ou qui craignent les contrôles de police, il n’est possible de se faire dépister que dans trois dispensaires une matinée par mois. Ce problème d’accès concerne également le suivi de soins des patients qui doivent obligatoirement se rendre au Centre hospitalier de Mayotte (CHM).

Pour atteindre les ODD 3 x 95, nous connaissons la voie à suivre à travers l’exemple des pays qui les ont déjà atteints par un engagement politique fort. Les éléments constitutifs d’une riposte efficace au sida à Mayotte seront le fruit d’investissements dans des axes prioritaires tels que le développement des campagnes de prévention à destination des habitants et la multiplication des lieux et créneaux de dépistage dans l’ensemble de l’île.

# Research article: Élimination de l'hépatite B en 2030 : Mythe ou réalité ?

**Published:** 2024-06-24

**Authors:** Victor de LÉDINGHEN

**Affiliations:** Centre expert hépatites virales Aquitaine (CEHVA), Hôpital Haut-Lévêque, Pessac, INSERM U1312, Université de Bordeaux), France

En 2014, l’Organisation mondiale de la santé a fixé pour objectif l’élimination des hépatites virales de la surface du globe d’ici 2030. L’élimination est définie par : une diminution de 90 % des nouvelles infections, associée à une réduction de la mortalité liée au virus de l’hépatite B (VHB) de 65 %; une prévalence de l’AgHBs < 0,1 % chez les enfants de moins de cinq ans; une couverture vaccinale > 90 %.

Pour atteindre cet objectif, plusieurs actions simultanées sont nécessaires : dépistage, traitement, prévention (vaccination).

Le dépistage universel n’est pas recommandé mais concerne toutes les personnes à risque :

- les personnes nées ou ayant résidé dans les régions de forte (Afrique subsaharienne, Asie) et moyenne endémicité (DROM-COM, Europe de l’Est et du Sud, Afrique du Nord, Moyen-Orient, souscontinent indien et Amérique du Sud);
- l’entourage proche et les partenaires sexuels d’une personne porteuse du VHB;
- les usagers de drogues par voie intraveineuse ou intra nasale;
- les patients susceptibles de recevoir des transfusions massives ou itératives;
- les voyageurs et les personnes amenés à résider dans un pays de forte ou moyenne endémicité;
- les adultes et enfants accueillis dans les institutions psychiatriques;
- les personnes séropositives pour le VIH ou le VHC ou ayant une infection sexuellement transmissible (IST) en cours ou récente;
- les personnes ayant un tatouage ou un piercing;
- les personnes séjournant ou ayant séjourné en milieu carcéral;
- les personnes ayant des relations sexuelles avec des partenaires différents;
- les personnes ayant un risque d’exposition professionnelle.

Le traitement de l’hépatite chronique virale B repose principalement sur l’utilisation au long cours d’un analogue nucléosidique (entécavir) ou d’un analogue nucléotidique (fumarate de ténofovir disoproxil).

Depuis 1982, il existe un vaccin efficace contre l’hépatite B. La primovaccination (deux injections suivies d’un rappel) est obligatoire pour tous les enfants nés à compter du 1^er^ janvier 2018. L’utilisation d’un vaccin combiné hexavalent contre la diphtérie, le tétanos, la coqueluche (vaccin acellulaire), la poliomyélite (vaccin inactivé), les infections à *Haemophilus influenzae* de type b et l’hépatite B permet d’immuniser les nourrissons contre ces maladies en une seule injection.

Les actions essentielles pour l’élimination de l’infection par le VHB sont : la sensibilisation des soignants et des politiques; la levée des stigmatisations; la vaccination; l’incitation à l’accès au diagnostic et au traitement; la surveillance épidémiologique; le renforcement des équipes existantes; l’encouragement de la recherche HBV cure.

# Research article: Réservoirs VIH et virus défectifs

**Published:** 2024-06-24

**Authors:** Héloïse DELAGRÈVERIE

**Affiliations:** Laboratoire de virologie, Centre hospitalier universitaire (CHU) Avicenne AP-HP, Bobigny.

Les VIH sont des rétrovirus, à génome ARN dans les virions et ADN dans les cellules infectées, dont le génome comporte à chaque extrémité une région « *Long Terminal Repeats* » (LTR) conservée chez toutes les souches virales et nécessaire à l’intégration dans le génome humain. Dans l’organisme, certaines cellules infectées ne sont pas détruites par l’infection mais persistent pour toujours en portant de l’ADN proviral capable de réactiver la réplication si le traitement antirétroviral est interrompu : c’est le réservoir viral. Le réservoir empêche la guérison, car il est maintenu inactif par les antirétroviraux mais non détruit.

Il n’existe pas de biomarqueur qui permette de différencier un T CD4+ « réservoir » d’un T CD4+ non infecté, et de nombreux tests de laboratoire ont été proposés pour estimer la fréquence des cellules infectées (ou « taille » du réservoir viral) dans l’organisme. Le test de référence est le *Viral Outgrowth Assay* dont le but est de quantifier les T CD4+ réactivables et producteurs de virus infectieux *ex-vivo*; il est techniquement lourd et coûteux et réservé à la recherche. Le test le plus accessible en France est la charge virale ADN VIH proviral, qui repose sur la quantification par PCR des régions LTR détectables dans un prélèvement de sang ou de tissu. Cette quantification surestime la taille réelle du réservoir viral car si les LTR sont nécessaires à l’intégration du génome viral, jusqu’à 98 % des provirus seraient criblés de mutations entre leurs LTR, porteurs de larges délétions ou d’autres aberrations génétiques et en réalité incapables de produire des virus : ce sont les provirus défectifs.

Chez un patient non virémique, un génotypage viral peut être obtenu par séquençage de l’ADN proviral. Réalisé sur du sang où les provirus sont d’autant plus rares que la durée de contrôle virologique a été longue, il peut manquer de sensibilité si l’échantillonnage proviral dans le prélèvement est trop faible pour le séquençage ou ne reflète pas l’archivage de tous les virus ayant répliqué au cours de l’histoire du patient. Il arrive aussi fréquemment que ce soit un provirus défectif par hypermutation qui se trouve analysé : le génotypage ne sera pas contributif, ou d’interprétation difficile en cas de mutations présentes sur des positions de résistance (cas célèbre de la M184I sur la transcriptase inverse, compatible mutation APOBEC).

Au total, le réservoir viral du VIH est constitué d’ADN proviral définitivement intégré dans le génome de l’hôte et qui persiste indéfiniment sous traitement, prêt à réactiver la réplication virale et la progression de l’infection en cas d’arrêt des antirétroviraux. Cet ADN proviral peut être utilisé pour le génotypage du virus au laboratoire, avec des performances (taux de réussite, sensibilité et spécificité) malgré tout inférieures au génotype classique sur virémie plasmatique. Si le dossier virologique du patient est accessible, une analyse cumulée des génotypes antérieurs sur échantillons virémiques sera plus contributive, ce qui souligne l’importance de documenter les échecs et rebonds virologiques.

# Research article: Inégalités sociales de santé : éléments de cadrage

**Published:** 2024-06-24

**Authors:** Cyrille DELPIERRE

**Affiliations:** Centre d’épidémiologie et de recherche en santé des populations (CERPOP) UMR1295, Inserm Université de Toulouse, France

Les inégalités sociales de santé (ISS) font référence à la relation étroite qui existe entre l’état de santé et la position socioéconomique et peuvent être définies comme des différences systématiques et observables d’état de santé entre individus de différents groupes sociaux. Les ISS ne concernent donc pas seulement les plus précaires mais bien la population dans son ensemble. Le gradient social de santé renvoie au fait que plus on s’élève dans la hiérarchie sociale, meilleur est l’état de santé, et inversement. Classiquement, ce gradient social de santé est mesuré par le revenu, le niveau d’éducation ou encore la profession. Mais on peut aussi citer d’autres déterminants sociaux moins utilisés comme le genre, l’origine ethno-raciale ou encore l’orientation sexuelle.

La France, malgré une espérance de vie à la naissance parmi les meilleures en Europe (85,2 ans pour les femmes; 79,2 ans pour les hommes), fait partie des pays où les inégalités sociales (IS) de mortalité sont les plus élevées, en particulier pour les hommes. Sur la période 2012-2016, chez les hommes, l’espérance de vie à la naissance des 5 % les plus aisés est de 84,4 ans, contre 71,7 ans parmi les 5 % les plus modestes, soit 13 ans d’écart (8 ans chez les femmes). Un homme cadre de 35 ans peut espérer vivre 47 ans dont 34 sans incapacité, tandis qu’un ouvrier peut espérer vivre encore 41 ans dont 24 sans incapacité. Ainsi, les catégories sociales moins favorisées font face à une « double peine » mêlant à la fois durée et qualité de vie. Ces données ne concernent que la France hors Mayotte, territoire pour lequel l’espérance de vie à la naissance est inférieure avec une différence moins marquée entre hommes et femmes (73,6 ans pour les femmes; 72,3 ans pour les hommes).

Pour expliquer ces inégalités, on peut distinguer trois grandes catégories de déterminants : le système de soins, les comportements, et les déterminants sociaux de la santé qui correspondent aux conditions sociales dans lesquelles les individus naissent, grandissent, vivent, travaillent et vieillissent. Ceux-ci conditionnent la façon dont une personne dispose des ressources physiques, sociales, économiques et personnelles pour satisfaire ses besoins, ses aspirations et s’adapter à son environnement. On distingue les déterminants physiques et matériels (expositions chimiques, physiques, spatiaux) et les déterminants psychosociaux (relations sociales, contraintes organisationnelles). Les déterminants sociaux et les comportements sont les déterminants essentiels de la santé et ont une influence majeure sur le risque de développer des maladies. Le système de soins a une influence importante mais non majoritaire sur la santé, principalement sur la prise en charge. Ainsi, les populations incorporent biologiquement, au sens littéral, le monde dans lequel elles vivent *via* des expositions physico-chimiques, psychosociales, comportementales et d’accès aux soins socialement distribuées. Les inégalités d’expositions physico-chimiques (pollution, qualité du bâti), comportementales (tabac, obésité) et psychosociales (expériences adverses) sont ainsi très marquées en France, et encore plus à Mayotte.

Pour lutter contre les ISS, il semble fondamental d’agir sur les conditions d’existence des individus. Les interventions et les politiques ne doivent pas se limiter aux déterminants intermédiaires (comportements par exemple), mais doivent inclure des politiques qui se concentrent spécifiquement sur les mécanismes produisant de façon systématique une distribution inégale des déterminants de la santé dans la population. En termes de type d’intervention, l’approche dite d’universalisme proportionné, semble être à privilégier. Elle met l’accent sur le fait que bien qu’universelle, l’intensité des efforts de politiques publiques et d’interventions pourrait être modulée en fonction des besoins des populations et de leur état de santé.

# Research article: Profil épidémique des personnes vivant avec le VIH dans les outre-mer : le grand écart d'un territoire ultramarin français à l'autre

**Published:** 2024-06-24

**Authors:** Loïc EPELBOIN, François BISSUEL, Karim ABDELMOUMEN, Aude LUCARELLI, Moncef MOUDHOIRE, Rémi MAYAN, Lam NGUYEN, Hassiba BELABBAS, Kevin DiALLO, Emilie FOLLENFANT, Adrien COURTOT, Victor PECOUL, Isabelle MONCHOTTE, Ludovic FLEURY, Claire BAIZET, Annick LAFFITTE, Clément COUTEAUX, Gaëlle BINAN, Cécile BROUARD, Florence LOT, Françoise Cazein, Erwan oEhLER, Mathieu NACHER, Isabelle LAMAURY, Catherine GAUD, Bénédicte SAUVAGE, André CABIÉ

**Affiliations:** Centre d’investigation clinique (CIC) Inserm 1424 Centre hospitalier de Cayenne, Guyane, France; Centre hospitalier Louis Constant Fleming, Saint Martin, France; Centre hospitalier de Mayotte, Mamoudzou, Mayotte, France; Corevih Guyane, Centre hospitalier de Guyane, France Association Narike M’sada, Mayotte, France; Direction de la santé, Polynésie française, France; Centre hospitalier François Dunan, Saint Pierre & Miquelon; Centre gratuit d’information, de dépistage et de diagnostic (CeGIDD) Sud, La Réunion, France; Centre hospitalier territorial Gaston-Bourret, Nouméa, Nouvelle-Calédonie, France; Direction provinciale de l’action sanitaire et sociale Province Sud, Nouvelle-Calédonie, France; Unité provinciale sanitaire et sociale - Dispensaire de Paita - Province Sud, Nouvelle-Calédonie, France; Bureau épidémiologie et statistiques - Service de Santé publique - DASS Nouvelle-Calédonie, France; Agence de Santé de Wallis et Futuna, France; Santé publique France, Saint Maurice, France; Centre hospitalier de Polynésie française, France; Coordination régionale de lutte contre le virus de l’immunodéficience humaine (Corevih) Guadeloupe, Saint Martin, Saint Barthélémy, Centre hospitalier universitaire (CHU) Pointe-à-Pitre, Guadeloupe, France Corevih océan Indien, Saint Denis de La Réunion, France Agence de communication Bcom, Cayenne, Guyane, France; Corevih Martinique, Centre hospitalier universitaire Pierre Zobda-Quitman, Martinique, France

## Contexte

Les départements et régions d’outre-mer (DROM : Guyane, Martinique, Guadeloupe, Mayotte, Réunion) et collectivités d’outre-mer (COM : Saint-Martin, Saint-Barthélémy, Saint-Pierre-et-Miquelon, Polynésie, Nouvelle-Calédonie et Wallis-et-Futuna) sont souvent assimilés à un ensemble homogène alors qu’ils sont très différents au plan sociogéographique et/ou culturel. L’objectif de ce travail était de décrire les caractéristiques épidémiologiques des personnes vivant avec le VIH (PVVIH) prises en charge dans ces territoires.

## Méthodologie

Les caractéristiques épidémiologiques ont été obtenues en consultant les données de Santé publique France pour les DROM, chaque direction des affaires sanitaires pour les autres, ainsi que les données des COREVIH. De plus, les principaux acteurs impliqués dans la surveillance et/ou le suivi des PVVIH ont été interrogés entre juin 2022 et janvier 2023, territoire par territoire.

## Résultats

La répartition du VIH est extrêmement hétérogène d’un territoire ultramarin à l’autre. Le taux d’incidence le plus élevé en 2021 est en Guyane (0,24 cas pour 1 000 habitants), suivie de Mayotte (0,21), la Guadeloupe (0,17) et la Martinique (0,15). Ces taux sont supérieurs au taux d’incidence le plus élevé observé au niveau hexagonal (0,08). La situation est plus modérée à La Réunion (0,04 nouveaux cas pour 1 000 habitants), et dans les îles du Pacifique (Polynésie, 0,05, et Nouvelle-Calédonie, 0,02). Saint-Pierre-etMiquelon et Wallis-et-Futuna ne rapportent pas de nouveaux cas en 2021, ni d’ailleurs de patients suivis localement.

La Guyane est le territoire français le plus touché avec une file active (FA) de 1 937 dans les trois hôpitaux et les centres de santé, mais entre 3 050 et 4 000 PVVIH perdues de vue, suivies en ville, parties ou décédées. La transmission est essentiellement hétérosexuelle en lien avec le multipartenariat et les rapports transactionnels, avec à peu près autant de femmes que d’hommes *(sex ratio* H/F (SR) 0,9). *A contrario,* en Martinique (FA ~ 1 134 PVVIH), comme à la Réunion (FA ~ 1 011), le profil des patients se rapproche de celui de l’hexagone : plus d’hommes, plus souvent contaminés suite à rapports homo/bisexuels, et une part de PVVIH nées à l’étranger beaucoup plus faible. Si la situation en Guadeloupe continentale (FA ~1 463) est intermédiaire (SR 1,7; 35 % des hommes contaminés lors de relations HSH et 38 % de PVVIH nées à l’étranger, celle à St-Martin, plus proche de celle de la Guyane (FA ~ 417) se distingue par une proportion majoritaire de femmes (SR=0,8), de transmission hétérosexuelle (86 %) et de PVVIH nées à l’étranger (80 %), essentiellement originaires de la Caraïbe. Dans l’océan Indien, alors que l’épidémie semble stabilisée à La Réunion, la situation est explosive ces dernières années à Mayotte, avec un nombre croissant de nouveaux diagnostics, principalement chez des personnes originaires des Comores, de Madagascar et de l’Afrique des Grands Lacs, et une transmission hétérosexuelle >90 % (FA ~ 342). Parmi eux, 9 % seulement des nouveaux patients sont mahorais et les patients étrangers représentent 80 % des nouveaux cas. Enfin, la situation dans les îles de l’océan Pacifique, Polynésie et NouvelleCalédonie, n’est ni comparable à celle des autres territoires, ni entre elles. En NouvelleCalédonie, dont la FA est de 273, l’isolement de l’île fait que les migrants ne représentent qu’une infime minorité des patients suivis, les deux principaux profils des patients suivis étant caricaturalement des patients HSH issus de l’hexagone d’une part, et une population kanake en situation de précarité essentiellement hétérosexuelle cisgenre d’autre part. Enfin, le suivi des patients de Polynésie française (FA ~ 170) est concentré sur le Centre hospitalier de Papeete, et la moitié des nouveaux cas annuels viennent de l’extérieur de la Polynésie française, principalement de France, souvent déjà diagnostiqués. Étonnamment, on ne dénombre aucun patient suivi pour l’infection par le VIH à Saint-Pierre-etMiquelon et à Wallis-et-Futuna.

## Conclusion

L’hétérogénéité des épidémies VIH dans les DROM/COM reflète des contextes socio-culturels et géographiques très différents entre ces territoires, ce qui rend nécessaire des approches ajustées, spécifiques, ciblées en matière d’offres de prévention et de soins.

# Research article: L'épidémie de VIH à Mayotte de 2019 à 2022 : un reflet des défis régionaux dans l'océan Indien

**Published:** 2024-06-24

**Authors:** Victor EIFERMAN, Karim ABDELMOUMEN

**Affiliations:** Unité des maladies infectieuses et tropicales, Centre hospitalier de Mayotte, Mamoudzou, Mayotte, France

## Introduction

Mayotte est le département le plus pauvre de France. En raison de sa situation géographique et de son histoire, ce territoire connaît une migration en provenance des îles voisines, notamment de l’archipel des Comores et de Madagascar, ainsi que de la région des Grands Lacs d’Afrique. En 2022, une augmentation significative des cas de VIH pris en charge a été observée. Cette étude analyse l’évolution épidémiologique du VIH à Mayotte de 2019 à 2022, en mettant l’accent sur les nouveaux cas diagnostiqués, les facteurs de précarité sociale et le contrôle de la charge virale sous traitement.

## Matériels et méthodes

Cette étude rétrospective reprend les patients diagnostiqués pour une infection VIH entre 2019 et 2022, et suivis en infectiologie au Centre hospitalier Mamoudzou de Mayotte. Les données cliniques, virologiques, immunologiques ont été recueillies, de même que les données démographiques et sociales comportant plusieurs déterminants sociaux de santé.

## Résultats

Entre 2019 et 2022, le nombre de nouvelles prises en charge pour une infection à VIH a presque doublé, passant de 66 en 2019 à 107 en 2022. La part de patients nouvellement diagnostiqués originaires de Madagascar a considérablement augmenté, passant de 25,6 % en 2019 à 45,3 % en 2022. Le nombre absolu de diagnostics chez les personnes originaires des Comores et de République démocratique du Congo a également augmenté. Les diagnostics pendant la grossesse, chez les travailleurs du sexe, et les co-infections avec d’autres infections sexuellement transmissibles ont augmenté.

De nombreuses différences ont été observées dans les caractéristiques de la population en fonction du lieu de naissance, notamment concernant le sexe, l’âge, les facteurs de risque spécifiques, la vulnérabilité, la gravité de l’infection, les parcours de soins et le contrôle de la charge virale sous traitement. D’importantes disparités existent entre les migrants et la population française, avec plus de 20 % des migrants n’ayant pas accès à l’eau potable ni à l’électricité, 40 % en insécurité alimentaire et 60 % ayant un revenu inférieur à 160 euros par mois.

L’analyse univariée a montré plusieurs facteurs présentant une tendance à un risque accru d’échec du traitement à six mois, notamment être de sexe féminin (p=0,051), être originaire de Madagascar (p=0,06) et être en situation irrégulière (p=0,069).

## Conclusion

Mayotte est désormais le département français présentant la plus forte incidence d’infection par le VIH. Cette augmentation est attribuée à une accélération de transmissions sur l’île, une migration sans soutien social adéquat et une offre de soins insuffisante. Il est essentiel de cibler les vulnérabilités, d’intensifier les efforts de prévention et d’élargir l’accès aux soins de santé pour contenir l’épidémie.

# Research article: Les enjeux de la santé sexuelle

**Published:** 2024-06-24

**Authors:** Christine KATLAMA

**Affiliations:** Service des maladies infectieuses et tropicales, Hôpital Pitié-Salpêtrière, Sorbonne Université, Paris, France

La notion de santé sexuelle fait partie intégrante de la santé depuis qu’en 2006 l’OMS en a défini très précisément les contours. Ainsi, la santé sexuelle est définie comme « un état de bien-être physique, émotionnel, mental et social en matière de sexualité, ce n’est pas seulement l’absence de maladie, de dysfonctionnement ou d’infirmité. La santé sexuelle exige une approche positive et respectueuse de la sexualité et des relations sexuelles, ainsi que la possibilité d’avoir des expériences sexuelles agréables et sécuritaires, sans coercition, ni discrimination et ni violence. Pour atteindre et maintenir une bonne santé sexuelle, les Droits Humains et Droits sexuels de toutes les personnes doivent être respectés, protégés et réalisés ». La santé sexuelle concerne chaque humain (homme ou femme), quels que soient son âge, sa couleur, sa race, son genre. Elle est universelle et essentielle. On peut considérer qu’il n’y a pas de vie épanouie sans santé sexuelle éclairée, pas de santé sexuelle sans éducation, pas de santé sexuelle sans liberté ni tolérance. Plusieurs fléaux doivent être reconnus et combattus :

- *Santé maternelle* : en 2020, 287 000 femmes sont mortes des suites de leur grossesse, d’un accouchement ou d’une IVG avec une mort maternelle toutes les deux minutes. Ces chiffres stagnent depuis 2016. 70 % des décès ont lieu en Afrique subsaharienne.
- *Le mariage des enfants* avant 18 ans constitue une violation des droits humains avec pour conséquences une scolarité réduite, l’exposition aux violences familiales, une mortalité maternelle plus grande.
- *Les mutilations sexuelles,* violation de droits humains, sont parfois officiellement interdites mais toujours perpétrées dans certains pays d’Afrique ou d’Asie. Elles concernent 200 millions de femmes dans le monde.
- *Les violences faites aux femmes* sont illustrées par des chiffres glaçants : 35 % des femmes ont subi des violences physiques et/ou sexuelles; en France, une femme en meurt tous les 2,5 jours; 41 % d’entre elles avaient subi des violences antérieures.

La santé sexuelle doit être enseignée, aux filles et aux garçons. Elle est l’affaire de chacun et de tous, soignants, éducateurs, parents, acteurs de santé publique, dans des structures dédiées à la prévention et le soin en santé sexuelle. Mais les individus sont aussi pris au piège des cultures et/ou des religions qui ont de tout temps développé des positions restées souvent archaïques, orchestrées fréquemment autour du pouvoir masculin, dans une binarité exclusive.

Gageons que l’éducation en santé sexuelle, à tous et pour tous, saura établir une nouvelle donne en santé basée sur la reconnaissance des droits, l’équilibre et l’harmonie.

# Research article: Vie sexuelle des personnes vivant avec le VIH : quelles spécificités ? Quelle approche sexologique ?

**Published:** 2024-06-24

**Authors:** Patrick PAPAZIAN

**Affiliations:** Service des maladies infectieuses et tropicales, Hôpital Pitié Salpêtrière, Hôpital Bichat, Paris, France

Les personnes vivant avec le VIH (PVVIH) ont statistiquement plus de risque d’être en mauvaise santé sexuelle que la population générale. Ce constat, fait depuis plus de dix ans dans les différents rapports français (Yeni puis Morlat), repose sur des enquêtes et études soulignant une activité sexuelle moins importante, notamment chez les femmes hétérosexuelles vivant avec le VIH (Vespa 2). Elles soulignent également une incidence élevée de dysfonctions sexuelles chez les PVVIH quel que soit leur sexe, tout particulièrement dysfonction érectile, diminution du désir, troubles du plaisir ou encore douleurs au moment des rapports sexuels. Globalement, on peut retenir qu’une PVVIH sur deux est en mauvaise santé sexuelle, ce qui est plus du double des chiffres observés en population générale. Des enquêtes plus récentes sont attendues (par exemple Vespa 3, et Moi Patient) pour actualiser ces données.

Les notions de TasP *(Treatment as Prevention,* le traitement comme prévention) et de « U=U » *(Undetectable = Untransmittable,* une personne vivant avec le VIH sous traitement efficace ne peut pas transmettre le virus) n’ont pas eu l’impact positif anticipé sur la sexualité de toutes les PVVIH, le poids du stigmate VIH/SIDA et des représentations attenantes restant lourd dans les sociétés : « la sexualité est un phénomène social » avant d’être médical.

Du point de vue de la prise en charge, aborder la sexualité, même brièvement en consultation, a un impact positif sur la santé sexuelle des PVVIH, en particulier rappeler la notion U=U comme y invitait le Lancet VIH en 2019 dans un plaidoyer.

Afin de favoriser cette discussion, les soignants (médecins, psychologues, infirmières…) et associatifs/médiateurs en santé doivent être formés à une approche sexologique en tenant compte des besoins spécifiques en fonction de leur lieu d’exercice, de leur culture, de leurs propres représentations. La construction collaborative d’un programme pédagogique est possible, comme en atteste l’initiative menée au Burundi depuis trois ans par l’association Sexologie sans frontières et son équipe franco-burundaise. C’est au prix d’une formation des acteurs du VIH à la prise en charge de la santé sexuelle et reproductive co-construite entre pays, répétée dans le temps et placée dans le registre non-jugeant du soin, que la santé sexuelle des PVVIH peut être améliorée durablement.

# Research article: Impact de la précarité et de la pauvreté sur le suivi des personnes vivant avec le VIH

**Published:** 2024-06-24

**Authors:** Nicolas VIGNIER

**Affiliations:** Hôpitaux universitaires Paris Seine-Saint-Denis, Hôpital Avicenne et Jean Verdier, AP-HP, Bobigny, France; Centre de recherche infection, antimicrobiens, modélisation, évolution (IAME), Inserm UMR 1137, Université Sorbonne Paris Nord, UFR SMBH, Bobigny, France; Institut Convergences et migration, Aubervilliers, France Centre d’investigation clinique (CIC) Inserm 1424, Centre hospitalier de Cayenne, Cayenne, Guyane, France

La précarité peut être définie comme une forte incertitude de conserver ou de récupérer une situation acceptable dans un avenir proche. C’est donc l’absence d’une ou de plusieurs sécurités comme un revenu propre, un logement, un soutien social, un titre de séjour pour les personnes migrantes, un accès à Internet, etc. qui entraine une insécurité plus ou moins étendue ayant des conséquences plus ou moins graves et définitives et pouvant conduire à la pauvreté et à une dégradation de l’état de santé. La précarité peut ainsi toucher tout le monde.

La pauvreté, quant à elle, peut se définir comme le fait de ne pas avoir assez d’argent pour subvenir à ses besoins de base. L’Insee, qui la dénomme privation matérielle et sociale, la définit comme le manque d’argent pour subvenir à au moins 5 des 13 besoins de base comme l’habillement, la nourriture, l’accès Internet, le paiement du loyer, se payer des vacances, pouvoir faire face à une dépense non prévue, remplacer ses meubles hors d’usage, boire un verre ou manger avec des amis, avoir une activité de loisir régulière, etc. Elle concerne 14 % de la population française et 77 % de la population à Mayotte. La grande pauvreté se définit, elle, comme l’association d’un revenu inférieur au seuil de pauvreté (900 € pour une personne seule dans l’hexagone) et des revenus insuffisants pour subvenir à au moins 7 des 13 besoins de base. Elle concerne 3 % de la population française.

Les inégalités sociales (et territoriales), qui peuvent être de santé, sont les écarts entre les personnes (ou les territoires), par exemple les plus aisées et les plus pauvres. Elles disent quelque chose des politiques sociales et des équilibres des sociétés. Par exemple à Mayotte, les 20 % des personnes les plus riches disposent d’un capital plus de 80 fois plus élevé que celui des 20 % les plus pauvres. C’est donc le territoire de France le plus inégalitaire.

La santé n’est pas étrangère à ces enjeux sociaux et est socialement fortement déterminée. Elle est ainsi conditionnée par les déterminants sociaux de la santé qui sont les facteurs structurels et les conditions de vie quotidiennes, à l’origine d’une grande partie des inégalités en santé entre pays et dans les pays (facteurs sociaux, politiques, économiques, environnementaux et culturels). On parle aussi de vulnérabilités qui rejoignent les composantes de la précarité et de la pauvreté, mais qui intègrent aussi la barrière de la langue, les discriminations subies, etc.

L’infection par le VIH est un cas d’école de maladie socialement déterminée à la fois dans son exposition, mais aussi dans le cadre de son suivi (perte de vue, difficultés d’accès aux soins, difficultés d’observance, peur d’être discriminé, etc.). Par exemple, une étude sur les perdus de vue dans l’Ouest guyanais a montré que près d’un tiers des patients avaient été perdus de vue en 10 ans et que les facteurs associés à cette perte de vue étaient un âge jeune, l’absence de couverture maladie, la précarité, une faible littératie en santé, le vécu de l’annonce, le lieu de résidence éloigné et l’absence d’initiation précoce d’un traitement. Elle a aussi la particularité, du fait de sa transmission sexuelle et de sa stigmatisation, de toucher plus spécifiquement les « populations clés » du VIH (travailleurs et travailleuses du sexe, hommes ayant des rapports sexuels avec des hommes, personnes trans, personnes migrantes, usagers et usagères de drogues par voie intraveineuse, personnes détenues et plus généralement les personnes en situation de vulnérabilité sexuelle).

Les leviers à notre disposition pour répondre à ces enjeux sont déjà d’appliquer et de faire appliquer le droit français, de lutter contre toutes les formes de discriminations en particulier en soins, de garantir un accès aux soins à tous sans reste à charge, de s’appuyer sur les organisations communautaires, de collaborer avec les personnels sociaux et éducatifs, et de promouvoir la création de postes de médiateurs en santé au sein de nos structures. Il est aussi utile de questionner nos services pour les adapter aux populations clés et aux personnes en situation de vulnérabilité, en recueillant et en tenant compte des déterminants sociaux de la santé. Il est également utile de former les soignants pour qu’ils montent en compétences culturelles et sociales, de travailler en réseau car on ne peut pas tout tout seul et d’aller vers les populations les plus éloignées du soin. Ces grands principes trouvent facilement un écho et une déclinaison dans le champ de la prévention et de la prise en soin de l’infection par le VIH qu’il nous incombe de décliner.

# Research article: L'enjeu du déploiement de la prophylaxie pré-exposition (PrEP) au VIH dans un contexte d'inégalités sociales de santé

**Published:** 2024-06-24

**Authors:** Nicolas VIGNIER

**Affiliations:** Hôpitaux universitaires Paris Seine-Saint-Denis, Hôpital Avicenne et Jean Verdier, AP-HP, Bobigny, France; Centre de recherche infection, antimicrobiens, modélisation, évolution (IAME), Inserm UMR 1137, Université Sorbonne Paris Nord, UFR SMBH, Bobigny, France; Institut Convergences et migration, Aubervilliers, France Centre d’investigation clinique (CIC) Inserm 1424, Centre hospitalier de Cayenne, Cayenne, Guyane, France

La prophylaxie pré-exposition (PrEP) au VIH est un nouvel outil de la prévention diversifiée de l’infection par le VIH. Le principe de la PrEP est de prendre un traitement (l’antirétroviral tenofovir-emtricitabine) avant d’être exposé à l’agent infectieux et, ainsi, d’éviter d’être infecté. Elle a démontré une efficacité très élevée en prévention du VIH (études HPTN, Iprex, Parteners Prep, Proudn ANRS-Ipergay, etc.), mais à condition que l’observance au traitement soit très élevée. Elle a aussi démontré un bon profil de tolérance, bien que le médicament puisse présenter une toxicité rénale et osseuse chez des personnes présentant des comorbidités. Elle est souvent prescrite en milieu spécialisé (service d’infectiologie ou de santé sexuelle), mais peut dorénavant être (primo)prescrite par tous les médecins. Plusieurs études montrent qu’elle reste largement méconnue, y compris au sein des populations clés de l’infection par le VIH, à l’exception des hommes ayant des rapports sexuels avec des hommes (HSH) qui représentent 97 % des 64 821 utilisateurs en France. Les premières études menées chez des femmes en Afrique subsaharienne ont eu du mal à démontrer une efficacité de la PrEP. Peu de personnes migrantes non HSH se sont vu prescrire la PrEP en France. Des travaux originaux récents, menés notamment auprès des travailleuses du sexe en Guyane (étude TDS Maroni) et en Île-de-France (Le Bus des femmes), ont permis de mettre en évidence plusieurs freins à son usage comme la difficulté à avaler le comprimé et à le prendre tous les jours, le risque de stigmatisation lié à la prise d’un antirétroviral, notamment en l’absence de domicile personnel, et des difficultés de compréhension et de littératie. Des études récentes menées en Afrique sont cependant encourageantes et ont démontré un impact sur le risque de s’infecter par le VIH de 76 % (étude SEARCH). La PrEP au VIH par injection mensuelle de cabotegravir qui sera bientôt disponible en France représente un espoir pour ces populations clés.

À Mayotte où résident plusieurs populations clés et où on a vu croître récemment le nombre de personnes vivant avec le VIH et les situations de vulnérabilité sexuelle et de travail du sexe, seules 15 personnes ont bénéficiées de la PrEP en 2022, dont 13 HSH et une personne trans. De même, la seule adresse renseignée sur les sites spécialisés pour les consultations PrEP est le CeGIDD/Centre de santé sexuelle du centre hospitalier de Mayotte, à Mamoudzou. On peut s’interroger sur la place de cet outil dans le contexte spécifique de Mayotte. Cependant, l’information en santé est un droit et toute personne résidante devrait pouvoir y avoir accès et se voir accompagner dans sa compréhension. À ce titre, une communication plus large sur l’ensemble des outils de la prévention diversifiée (PrEP, traitement post exposition (TPE), dépistage élargi notamment en aller vers, lutte contre les violences sexuelles faites aux femmes et aux hommes, etc.) mériterait d’être déployée au-delà des efforts récents de communication sur l’usage des préservatifs. L’offre de lieux de consultation où l’on peut se voir prescrire la PrEP et le TPE doit être renforcée et être suffisamment visible pour être identifiée par ceux qui en ont besoin. Il faut cependant s’attendre à un cumul de nombreux freins à son utilisation (informationnels, sociaux, culturels, etc.). Cela invite à mener au préalable une réflexion approfondie avec les acteurs communautaires du territoire sur la meilleure approche.
